# The proteomic landscape of extracellular vesicles derived from human intervertebral disc cells

**DOI:** 10.1002/jsp2.70007

**Published:** 2024-11-05

**Authors:** Li Li, Hadil Al‐Jallad, Aiwei Sun, Miltiadis Georgiopoulos, Rakan Bokhari, Jean Ouellet, Peter Jarzem, Hosni Cherif, Lisbet Haglund

**Affiliations:** ^1^ Department of Surgery, Division of Orthopaedics McGill University Montreal Quebec Canada; ^2^ The McGill Scoliosis and Spine Group, McGill University Health Centre Montreal Quebec Canada; ^3^ The Orthopaedic Research Laboratory, Research Institute of the McGill University Health Centre Montreal Quebec Canada; ^4^ Shriners Hospital for Children Montreal Quebec Canada; ^5^ Department of Anatomy and Cell Biology McGill University Montreal Quebec Canada; ^6^ Department of Surgery, Division of Neurosurgery Faculty of Medicine, King Abdulaziz University Jeddah Saudi Arabia

**Keywords:** different degeneration grades, extracellular vesicles, human intervertebral disc, low back pain, proteomic cargo

## Abstract

**Background:**

Extracellular vesicles (EVs) function as biomarkers and are crucial in cell communication and regulation, with therapeutic potential for intervertebral disc (IVD)‐related low back pain (LBP). EV cargo is often affected by tissue health, which may affect the therapeutic potential. There is currently limited knowledge of how the cargo of IVD cell‐derived EVs varies with tissue health and how differences in proteomic profile affect the predicted biological functions.

**Methods:**

Our study purified EVs from human IVD cell conditioned media by size‐exclusion chromatography. Nanoparticle tracking analysis was conducted to measure EV size and concentration. Transmission electron microscopy and Western blot were performed to examine EV structure and markers. Tandem mass tag‐mass spectrometry was conducted to determine protein cargo.

**Results:**

Most EVs were exosomes and intermediate microvesicles with an increasing amount linked to disease progression. Of the proteins detected, 88.6% were shared across the non‐degenerate, mildly‐degenerate, and degenerate samples. GO and KEGG analyses revealed that cargo from the mildly‐degenerate samples was the most distinct, with the proteins in high abundance strongly associated with extracellular matrix (ECM) organization and structure. Shared proteins, highly expressed in the non‐degenerate and degenerate samples, showed strong associations with cell adhesion, ECM–receptor interaction, and vesicle‐mediated transport, respectively.

**Conclusions:**

Our findings indicate that EVs from IVD cells from tissue with different degrees of degeneration share a majority of the cargo proteins. However, the level of expression differs with degeneration grade. Cargo from the mildly‐degenerate samples exhibits the most differences. A better understanding of changes in EV cargo in the degenerative process may provide novel information related to molecular mechanisms underlying IVD degeneration and suggest new potential treatment modalities for IVD‐related LBP.

## Graphical abstract

Extracellular vesicles (EVs) derived from human intervertebral disc cells present markers CD9, CD81, CD82, ALIX, FLOT1/2, TSG101, and ANXA5 with differential expression levels linked to the degenerative grade of the tissue. The proteomic cargo is majorly shared but the expression levels vary with degeneration grade. The differentially expressed proteins are associated with (1) cell adhesion and ECM–receptor interaction, (2) ECM organization and structure, and (3) vesicle‐mediated transport in the non‐, mildly‐, and degenerate samples, respectively.

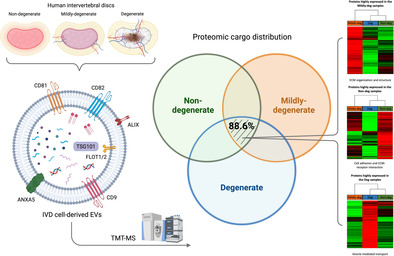

## 1 INTRODUCTION

Intervertebral disc (IVD) degeneration is a contributor to low back pain.^1^, ^2^, ^3^ According to the *Global Burden of Diseases, Injuries, and Risk Factors Study* in 2019,^4^ low back pain was in the top 10 causes of the disability‐adjusted life‐years for the 10–24‐year, 25–49‐year, and 50–74‐year age groups. There is currently no disease‐modifying treatment for IVD‐related low back pain. Human mesenchymal stem cell (MSC)‐based therapy has been intensively investigated as a potential therapy. MSCs given the appropriate conditions can differentiate into a nucleus pulposus (NP)‐like phenotype. MSCs can also release trophic factors with immunomodulatory and anti‐inflammatory therapeutic potentials.^5^ However, MSC‐based preclinical and clinical trials present variable outcomes, which may be due to poor cell engraftment, viability, and retention of the implanted cells in the harsh IVD environment. The studies vary greatly in terms of cell type, the number of transplanted cells, and the delivery system.^2^, ^6^, ^7^

Cell‐free supplementation strategies, utilizing the trophic factors found in extracellular vesicles (EV), are now being investigated as an alternative treatment approach for IVD‐related low back pain^3^, ^6^, ^7^, ^8^, ^9^ as they demonstrate a similar or greater therapeutic effect than MSCs. They have fewer ethical and regulatory concerns and have greater accessibility and longer preservation periods as an off‐the‐shelf product.^6^, ^10^, ^11^ EVs are lipid bilayer membrane‐delimited particles released by all cell types.^12^ They are classified depending on size and molecular composition into exosomes (30–150 nm),^6^ microvesicles (100–1000 nm),^13^, ^14^ and apoptotic bodies (1–5 μm).^13^ EVs carry cargo, including proteins, nucleic acids, and lipids.^15^ The EV cargo is transferred to neighboring cells and conveys important molecular messages that affect the recipient cell's phenotype and function. The cargo is suggested to be influenced by the state of the parental cells but it is challenging to compare separate studies as culturing and purification conditions that affect the cargo are not standardized.^6^, ^12^, ^14^, ^15^, ^16^

Parameters including cell isolation, expansion, and culture conditions can affect EV production and cargo. The isolation and expansion of cells may select for more proliferative cell populations, including progenitor cells, which are known to reside within tissues.^17^, ^18^, ^19^, ^20^ EV biogenesis genes are upregulated in 3D compared to 2D culture systems.^21^ 3D cultures of hydrogels, scaffolds, or spheroid culture systems provide a more physiological environment that more accurately replicates the extracellular matrix and mechanical environment.^22^, ^23^ Some 3D cultures have been observed to increase EV secretion^24^ with reduced size^25^ and enhanced more potent cargo.^26^ Mechanical loading is suggested to influence EV production and cargo composition. Hao et al. demonstrated that mechanical stimulation, such as mechanical squeezing using microfluidic devices, increased the secretion of small EVs from human bone marrow‐derived MSCs.^27^ Biomechanical forces, such as stretching, compression, and shear stress, affect EV quantity and cargo in a context‐dependent manner.^28^, ^29^ Oxygen levels also affect EVs with increased release of miRNAs and proteins reflective of a hypoxic response.^30^, ^31^ However, it is not known if hypoxia results in EVs with a greater regenerative potential. It is suggested that glucose levels can influence EV amount and content. High glucose concentrations can enhance the secretion of especially small EVs in human placental cells, highlighting a potential link between glucose levels and EV dynamics.^32^ Another study indicated that insulin resistance increased EV secretion and affected the cargo, particularly proteins related to insulin signaling.^33^ In addition, engineered EVs represent a promising therapeutic strategy for IVD pathology, particularly due to their ability to deliver specific molecular cargo that modulates cellular functions. It has been shown that EVs derived from human umbilical cord MSCs can regulate key molecular pathways in IVD degeneration, such as inhibiting ferroptosis in mouse NP cells through the *MALAT1*/*miR‐138‐5p*/SLC7A11 regulatory network.^34^ Additionally, engineered EVs have been utilized for non‐viral delivery of the transcription factor gene *FOXF1*, and restore the tissue function while modulating pain behavior in a mouse model.^35^ The effect of different culture systems, mechano‐stimulation, glucose, and oxygen levels on IVD cell‐derived EVs is currently not well established, and it is not clear if the regenerative potential is affected by these factors.

Although most studies have investigated EVs from MSCs, some studies have reported the regenerative potential of EVs derived from IVD cells.^1^, ^36^, ^37^, ^38^, ^39^, ^40^ However, no previous study has systematically investigated the cargo of EVs derived from human IVD cells isolated from tissue with different degrees of degeneration, while using the same culturing and purification conditions. Understanding how the proteomic cargo differs as a consequence of tissue health, culturing conditions, and other factors may guide in selecting the best source for the development of future targeted therapies.

The objective of this study was to characterize EVs derived from human IVD cells that were isolated from tissue with different degeneration grades. To minimize the effects of isolation and expansion in monolayer cultures, we only used cells in passage 1 at a constant glucose level of 2.25 g/L in this study. We evaluated the size, EV markers, and proteomic profile with tandem mass tag‐mass spectrometry and used bioinformatics for functional annotation prediction and pathway analysis.

## 2 MATERIALS AND METHODS

### 2.1 Study workflow

**FIGURE 1:**
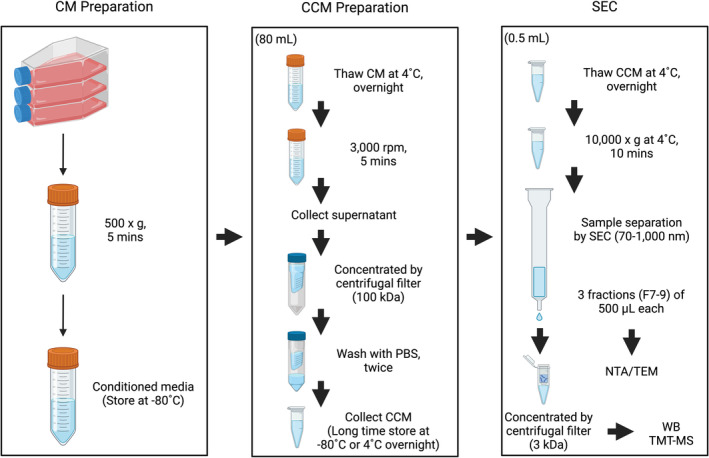
Schematic overview of the experimental workflow. Study workflow including conditioned media (CM) preparation, extracellular vesicle (EV) isolation and purification using centrifugation and size‐exclusion chromatography (SEC), and EV characterization by Western blot, nanoparticle tracking analysis (NTA), transmission electron microscopy (TEM), and tandem mass tag‐mass spectrometry (TMT‐MS).

A rapid, simple, and cost‐effective protocol was established for the enrichment, separation, and purification of EVs from human IVD cell conditioned media (CM).^41^, ^42^, ^43^, ^44^ CM from days 0–4 and 5–8 was collected and pooled, followed by centrifugation and size‐exclusion chromatography (SEC). Fractions 7–9 were further characterized by nanoparticle tracking analysis (NTA), transmission electron microscopy (TEM), and tandem mass tag‐mass spectrometry (TMT‐MS) (Figure 1).

### 2.2 IVD tissue isolation and cell culture

#### 2.2.1 Tissue isolation

IVD tissue was procured from consenting organ donors as well as patients undergoing spinal surgical interventions for low back pain. All tissue collections were conducted in strict compliance with ethical protocols approved by McGill University's Institutional Review Board (IRB# 2019‐4896). Donor and patient demographic information are delineated in Table 1. The IVDs were graded according to the visual Thompson grade and were divided into three groups: Grades 1–2, as Non‐deg, Grade 3, as Mildly‐deg, and Grade 4–5, as Deg. NP and inner annulus fibrosus (AF) tissue were retrieved. Subsequent cell isolation was performed as previously published.^45^ In brief, the tissue was rinsed, minced, and digested using 200 U/mL type II collagenase (ThermoFisher Scientific, Toronto, ON, Canada) for 16 h at 37°C. The isolated cells were washed and resuspended in Dulbecco's Modified Eagle Medium (DMEM) (Sigma‐Aldrich, Oakville, ON, Canada) containing 2.25 g/L glucose. The glucose level was selected for consistency with the EV collection media. The media was further supplemented with 10% (v/v) fetal bovine serum (FBS), 50 μg/mL Gentamicin, 2 mM GlutaMAX™, and 50 mM HEPES (ThermoFisher Scientific, Toronto, ON, Canada) and incubated at 37°C in a 5% CO_2_ environment.

**TABLE 1:** Donor and patient information and applications.

Donor	Age	Sex	Cause of death/disc pathology	IVD levels	Thompson grade	Experiments				
						EV^1^ purification	Western blot	TEM^2^	NTA^3^	TMT‐MS^4^
Organ donors										
1	18	M	Trauma	L3‐S1	1, Non‐degenerate	✔		✔	✔	✔
2	19	M	Drug overdose	L3‐S1	1, Non‐degenerate	✔			✔	✔
3	55	M	Fell down	L3‐4	2, Non‐degenerate	✔		✔	✔	✔
4	21	M	Motor vehicle accident	L2‐5	1, Non‐degenerate	✔			✔	✔
5	16	M	Drug overdose	L2‐3 & L4‐S1	1, Non‐degenerate	✔				
6	57	F	Anoxia	T12‐L2 & L3‐4	2‐3, Mildly‐degenerate	✔		✔	✔	✔
7	60	F	Brain hemorrhage	L3‐S1	3, Mildly‐degenerate	✔			✔	✔
8	54	F	Stroke	T11‐L2	1 & 3, Mildly‐degenerate	✔			✔	✔
9	63	M	Cranial trauma	L2‐5	2‐3, Mildly‐degenerate	✔			✔	✔
10	68	M	Brain hemorrhage	L4‐S1	3, Mildly‐degenerate	✔		✔	✔	✔
11	78	M	Unknown	L3‐5	3‐4, Degenerate	✔			✔	✔
Surgical samples										
1	32	M	Simple herniation	L4‐S1	3, Mildly‐degenerate	✔			✔	✔
2	32	M	Spinal stenosis/Spondylo	L5‐S1	3‐4, Degenerate				✔	
3	24	M	Simple herniation	L4‐5	4, Degenerate	✔	✔		✔	✔
4	45	F	Simple herniation	L4‐S1	4, Degenerate	✔	✔		✔	✔
5	50	F	Spinal stenosis/Spondylo	L5‐S1	4‐5, Degenerate	✔	✔			
6	40	F	Simple herniation	C6‐7	4‐5, Degenerate	✔	✔			

Abbreviations: 1 EV, extracellular vesicle; 2 TEM, transmission electron microscope; 3 NTA, nanoparticle tracking analysis; 4 TMT‐MS, tandem mass tag‐mass spectrometry.

#### 2.2.2 Cell culture

Isolated cells were seeded at 1 million cells per T75 cell culture flask (Sarstedt, TC Flask T75, Stand, Vent. Cap, Germany). For cultures derived from organ donor tissues, NP to inner AF cells were combined at a 1:1 ratio. While cells from surgical samples were obtained from a mixture of NP and inner AF tissue. Cells were cultured in DMEM media as described above. The cultures were maintained at 37°C in a 5% CO_2_ environment. The culture media was refreshed twice per week until they reached ~90% confluence in passage 1. To minimize the effects of isolation and expansion in monolayer cultures, we only used cells at passage 1 in this study.

### 2.3 EV‐depleted FBS preparation

EV‐depleted FBS (dFBS) was prepared in our laboratory with modifications of a previously published protocol.^46^ Heat‐inactivated standard FBS was subjected to centrifugation for 5 min at 3000 rpm at room temperature (RT). The supernatant was then passed through a 0.22 μm pore‐sized filter (Cat. #S2GPU01RE, Millipore, Burlington, MA, USA). The filtered FBS was centrifuged at 8400 rpm (g‐force 12 091 g_max_ and 8693 g_average_, k‐factor 2961), for 30 min at 4°C. The collected supernatant was subsequently centrifuged at, 26400 rpm (g‐force 119 431 g_max_ and 85 865 g_average_, k‐factor 300) for 70 min at 4°C. Only the lighter‐hued top fractions of the supernatant, approximately constituting the top 9/10 of the total volume, were retained and used for supplementation in the cell culture media. Both centrifugation steps used an SW32 Ti swinging‐bucket rotor, the Open‐Top Thinwall Ultra‐Clear™ tubes, and the Optima™ XPN‐80 Ultracentrifuge (Cat. #369694, #344058, and #A95765, Beckman Coulter Canada, Inc., Montreal, QC, CA).

### 2.4 Conditioned media (CM) collection for EV enrichment and cell lysate preparation

Passage 1 cells were allowed to expand for 72–96 h to reach about 90% confluency. The expansion media was removed, and cells were washed three times 1 min with PBS, at 37°C. Subsequently, the media were replaced with freshly prepared EV collection media (low‐particulate, xeno‐free, and protein‐free), specifically, RoosterCollect™‐EV media (Cat. #M2001, RoosterBio, Inc., Frederick, MD, USA), with a glucose level of 2.5 g/L, supplemented with 1% dFBS. CM was retrieved after 4 days, fresh EV collection media was added and collected after an additional 4 days. For host cell lysate preparation, disc cells were detached using a 0.25% trypsin solution and lysed in RIPA lysis buffer (ThermoFisher Scientific, Toronto, ON, Canada). The CM was centrifuged at 500*g* for 5 min at RT to eliminate cellular debris. The supernatant was carefully separated and stored at −80°C for future applications, as a previous study has shown that storing samples at −80°C for up to a month can effectively maintain the quality and quantity of EVs.^47^

### 2.5 EV‐enriched concentrated CM (CCM) preparation

CM was thawed at 4°C overnight and centrifuged at 3000 rpm for 5 min at RT to remove any precipitation. The clarified media were then subjected to further centrifugation using a Sorvall LYNX 4000 Superspeed Centrifuge equipped with a Fiberlite™ F21‐8 × 50y Fixed‐Angle Rotor (Cat. #75006580 and #096‐084019, ThermoFisher Scientific, Toronto, ON, Canada) at 8400 rpm for 30 min at 4°C. Following this, the supernatant was carefully collected and concentrated using an Amicon® Ultra‐15 Centrifugal Filter Unit (molecular weight cut‐off: 100 KDa, Cat. #UFC510096, Millipore, Burlington, MA, USA) according to the manufacturer's instructions. Each filter unit was rinsed using sterile PBS and clarified CM was concentrated by centrifugation at 5000*g* for 35 min at 4°C. This process was repeated until all the CM had been processed. Subsequently, the CM was replaced with PBS and processed twice for centrifugation. The EV‐enriched CCM was stored at −80°C for long‐term preservation or at 4°C overnight if processing was scheduled for the next day, as a previous study has shown no significant differences in EV quantity or quality between EVs isolated from samples stored at 4°C and −80°C for up to 7 days.^48^

### 2.6 Purification of CCM with size‐exclusion chromatography (SEC)

For the purification process, clarified CCM was applied to a qEVORIGINAL GEN 2 size‐exclusion column (Izon Science, Ltd., Addington, Christchurch, New Zealand). The EVs were eluted in 500 μL fractions according to the manufacturer's instructions. Fractions rich in particles but low in protein content (fractions 7–9) were pooled. The pooled fractions were then further concentrated using Amicon® Ultra‐0.5 Centrifugal Filter Units (molecular weight cut‐off: 3 KDa, Cat. #UFC500396, Millipore, Burlington, MA, USA), yielding a final volume of 80 μL, for downstream applications.

### 2.7 Quantification of protein content

Protein concentration was determined using the Micro BCA™ Protein Assay Kit (ThermoFisher Scientific, Toronto, ON, Canada) as per the manufacturer's guidelines and a TECAN Infinite M200 PRO plate reader equipped with i‐control 1.9 Magellan software (TECAN, Männedorf, Switzerland).

### 2.8 Western blot analysis

#### 2.8.1 Sample preparation

Purified and concentrated EVs, and soluble protein samples, were mixed with 4× NuPAGE™ LDS Sample Buffer (ThermoFisher Scientific, Toronto, ON, Canada), with 10% (v/v) β‐mercaptoethanol for ANAX5, FLOT1, Tom20, and 58K Golgi or without for CD81. The samples were heated at 95°C for 5 min. The processed samples were applied to 1.0 mm Novex™ 4%–20% Tris‐Glycine Mini Protein Gels (ThermoFisher Scientific, Toronto, ON, Canada). The separated proteins were then transferred to nitrocellulose membranes (GE Healthcare Canada, Mississauga, ON, CA). The membranes were blocked with 5% bovine serum albumin in Tris‐buffered saline supplemented with 0.1% Tween‐20 (TBS‐T; Sigma‐Aldrich, Oakville, ON, Canada) followed by 5% skimmed milk in TBS‐T and exposed to the primary antibodies (Table 2) ANXA5, FLOT1, CD81, and Tom20 (Abclonal, Woburn, MA, USA) and 58K Golgi (Abcam, Toronto, ON, CA). After washing, the membranes were exposed to secondary antibodies (Table 2) and visualized with Immobilon ECL Ultra Western HRP Substrate (EMD, Inc., Mississauga, ON, CA), and the ImageQuant™ LAS 4000 imaging system (GE Healthcare Canada, Mississauga, ON, CA).

**TABLE 2:** Antibody information.

Protein of interest	Molecular weight (KDa)	EV sample loading weight (μg)	Associated positive control sample loading weight (μg)	Primary antibody dilution	Primary antibody Cat. #	Secondary antibody dilution	Secondary antibody Cat. #
ANXA5	35–36	20	20	1/5000	A13945	1/10000	ab6721
FLOT1	47–48	20	20	1/2000	A3023	1/5000	ab6721
CD81	22–25	40	10	1/1000	A5270	1/5000	ab6721
TOM20	16	20	20	1/1000	A19403	1/5000	ab6721
58 K Golgi	58	20	20	1/1000	ab27043	1/5000	ab6789

### 2.9 Transmission electron microscopy (TEM) analysis

An AGS160 Carbon Films on 200 Mesh Grids Copper (cc grid) (Agar Scientific, Ltd., Stansted, Essex, UK) was prepared using a PELCO easiGlow™ Glow Discharge Cleaning System (TED PELLA, Inc., Redding, CA, USA). The system was operated at 20 mA current and an air pressure of 0.39 mBar for 20 s to achieve effective coating. The purified EV fractions were diluted 1:100 in PBS that had been pre‐filtered through a 0.22 μm membrane and 20 μL was deposited as a drop on parafilm. A coated cc grid was placed on the drop. The immobilized EVs were fixed in 2.5% (v/v) glutaraldehyde for 2 min and rinsed with PBS for 1 min. Negative staining was performed with a 2% (w/v) uranyl acetate solution, which was applied for 1 min followed by three PBS washes, for 1 min. The samples were carefully blotted dry using absorbent Waterman paper and left to air dry for 5 min at RT. The samples were subjected to high‐resolution imaging using a Talos F200X 200KV TEM (ThermoFisher, Toronto, ON, Canada).

### 2.10 Nanoparticle tracking analysis (NTA)

NTA was conducted to quantify particle numbers and size distribution. All analyses were performed using NanoSight NS300 instrument, paired with NanoSight NTA 3.4 software (Malvern Panalytical, Ltd., Malvern, UK). Camera settings were calibrated, with levels set at 13 for Fraction 7 and 14 for Fractions 8 and 9 (shutter/ms 30, slider gain 125). The detection threshold was set at level 5, and automatic blur and minimum track length were enabled as post‐acquisition settings. Particle‐free PBS was used to dilute samples before measurement. An initial dilution factor of 5 was employed and subsequently adjusted for each sample to achieve a particles‐per‐frame range of 20–100 and a total particle count between 1 × 10^8^/mL and 1 × 10^9^/mL. A 1 mL syringe was used to load each fraction sample into the sample chamber. The camera focus was adjusted so that particles appeared as distinct, sharp points of light. This setting was maintained consistently across all samples. For each sample, five 30‐s video recordings were captured, separated by 10‐s intervals. Data were analyzed by the NanoSight NTA 3.4 software.

The DNA concentration of the host cells was measured using the Hoechst method as previously described.^49^ The particle concentration of the samples was normalized to the total DNA quantity of the host cells and (particles/mL)/ng DNA was presented.

### 2.11 Tandem mass tag–mass spectrometry (TMT‐MS) and analytical procedures

#### 2.11.1 Mass spectrometry

EV fractions were loaded onto a single stacking gel for purification. The gel band was reduced with dithiothreitol, alkylated with iodoacetic acid, and digested with trypsin. The generated peptides were labeled using the TMTpro™ 16plex Label Reagent, as specified by the manufacturer. Labeled peptides were separated into eight fractions via a Pierce™ High pH Reversed‐Phase Peptide Fractionation Kit. Each fraction was reconstituted in 0.1% (v/v) aqueous formic acid, and an aliquot containing 2 μg was introduced onto a Thermo Acclaim Pepmap pre‐column (75 μM ID × 2 cm C18 3 μM beads). The peptide mixture was then separated using an Acclaim Pepmap Easyspray analytical column (75 μM × 15 cm with 2 μM C18 beads) on the Dionex Ultimate 3000 UHPLC System at a flow rate of 250 nL/min. A linear gradient of 2%–35% organic solvent, compounded with 0.1% (v/v) formic acid in acetonitrile, was applied over a 3 h runtime.^50^ An Orbitrap Fusion instrument was operated in data‐dependent acquisition‐multi‐stage mass spectrometry 3 (DDA‐MS3) mode using standardized settings for MS3‐level SPS TMT quantification (all reagents and equipment sourced from ThermoFisher Scientific, Toronto, ON, Canada). MS1 scans were acquired at a resolution of 120 000, covering an *m/z* range from 375 to 1500, with ion collection continuing for 50 ms or until reaching the automatic gain control (AGC) target of 4 × 10^5^. Precursors with charge states ranging from 2 to 5 were designated for MS2 analysis, isolated within an *m/z* window of 0.7, and fragmented through collision‐induced dissociation at 35% energy, which was then read out on the linear ion trap in rapid mode. The top 10 (by height) sequential precursor notches extracted from MS2 spectra were designated for MS3 TMT reporter ion quantification. These were isolated within an *m/z* window of 2 and subjected to fragmentation via higher‐energy collisional dissociation at 65% energy. The fragment ions were subsequently detected in the Orbitrap at a resolution of 60 000 with a maximum ion injection time of 105 ms or until an AGC target of 1 × 10^5^ was reached.^51^

#### 2.11.2 Data analysis

Raw data files (.raw) were processed for protein identification and TMT reporter ion quantification using Proteome Discoverer 3.0 (ThermoFisher Scientific, Toronto, ON, Canada). Spectra were aligned against the human protein fasta database, as procured from UniProt (2022 version). Dynamic modifications were configured for oxidation (M) and *N*‐terminal acetylation. Carbamidomethyl cysteine was defined as a static modification, along with TMT tagging at both peptide N‐termini and lysine (K) residues. The outcome was filtered to a 1% false discovery rate (FDR) criterion.^51^

### 2.12 Statistical analysis and illustration

Statistical analyses were performed by GraphPad Prism 10 (version 10.0.2 (171)) (GraphPad Software, Inc., La Jolla, CA, USA) using Welch's ANOVA test with Dunnett's T3 multiple comparisons test. All data are not inconsistent with a Gaussian distribution. *p* < 0.05 was considered statistically significant. All assessments were conducted with three or more independent experiments, indicated by “*n*” in the figure legends.

Processes/pathway analyses were performed by ShinyGO 0.80 (http://bioinformatics.sdstate.edu/go80/) and STRING database with default settings. Venn diagrams were generated by Venny 2.1 (https://bioinfogp.cnb.csic.es/tools/venny/). Data are presented as mean ± SEM. The schematic illustrations (graphical abstract and Figure 1) were created with BioRender.com (accessed on July 23, 2024, publication license numbers: #RF273H3YZF and #RT273H340Z).

## 3 RESULTS

### 3.1 Characterization of human IVD‐cell‐derived EVs

**FIGURE 2:**
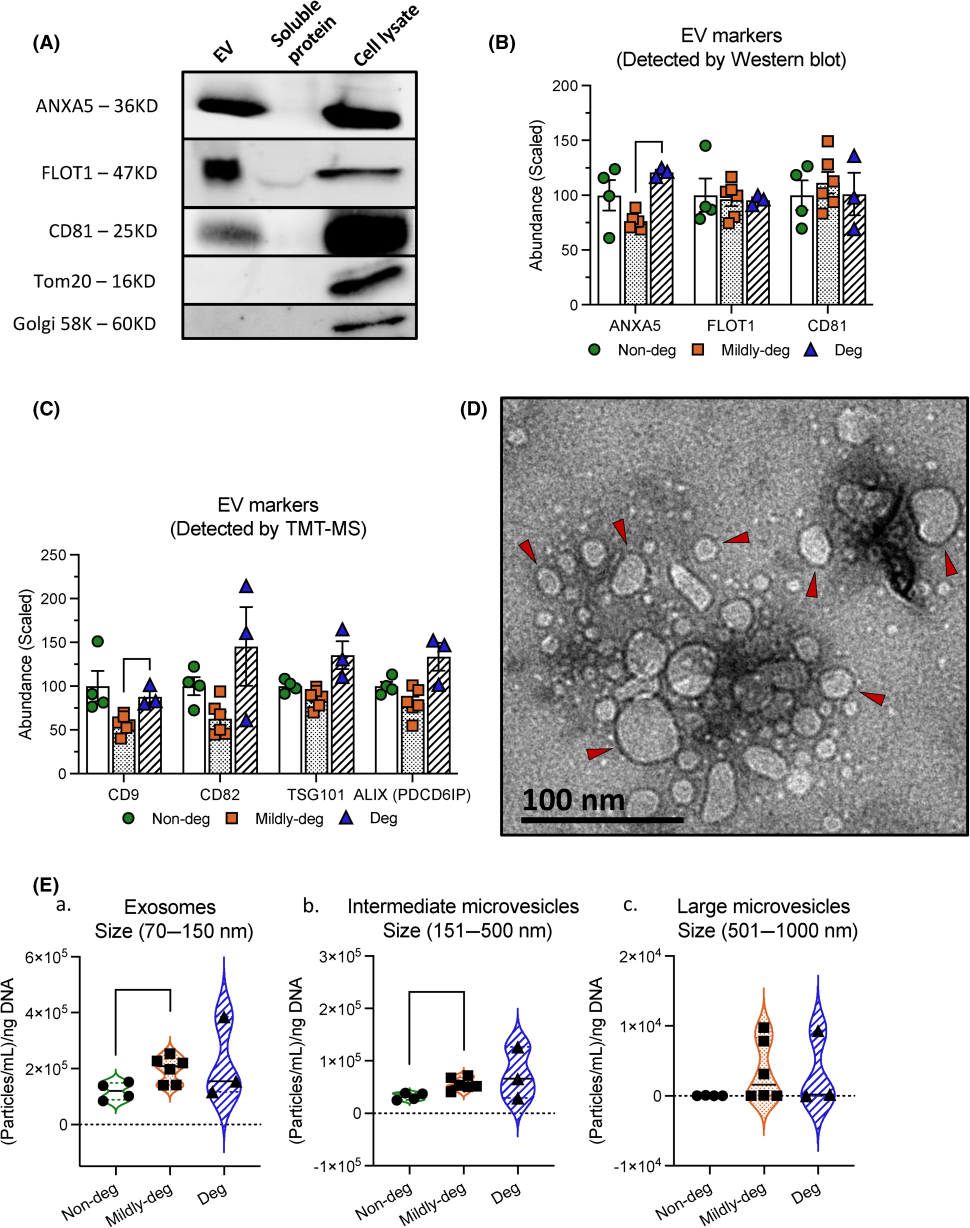
Characterization of EVs derived from human IVD cells. A. Western blot determining the presence of the EV markers ANXA5, FLOT1, and CD81 and the absence of the mitochondria marker TOM20 and the Golgi protein 58K Golgi. *n* = 4. B. TMT‐MS analyses confirmed the presence and relative expression of the EV markers ANXA5, FLOT1, and CD81. *n* = 3–6. C. TMT‐MS analyses show the relative expression of the additional EV markers CD9, CD82, TSG101, and ALIX (PDCD6IP). *n* = 3–6. D. TEM of purified IVD‐cell‐derived EVs (red triangle). *n* = 4. Scale bar = 100 nm. E. NTA showing the concentration and size distribution of EVs: (a) exosomes (70–150 nm), (b) intermediate microvesicles (151–500 nm), and (c) large microvesicles (501–1000 nm). *n* = 3–6. Values are presented as mean ± SEM. * and ** indicate statistically significant changes assessed by Dunnett's T3 multiple comparisons under Welch's ANOVA tests: *p* < 0.05 and *p* < 0.01.

Western blot analysis was performed to validate the purification procedure. The typical EV markers ANXA5, FLOT1, and the tetraspanin CD81 were detected in the EV fraction and cell lysate, but not in the soluble protein fraction (Figure 2A), confirming a successful separation of EVs and soluble proteins. In addition, the mitochondria and Golgi markers TOM20 and Golgi 58K were not detected in the EV fraction or the soluble protein fraction but were detected in the cell lysate (Figure 2A), confirming a successful exclusion of these organelles from the purified EV samples. TMT‐MS analysis was performed to establish the proteomic landscape of cell‐derived EVs from IVD tissue with an increasing degree of degeneration. The presence of ANXA5, FLOT1, and CD81 was confirmed in the TMT‐MS analysis where the relative expression (scaled abundance) of ANXA5 was elevated in Deg samples and reached significance when compared to Mildly‐deg IVD tissue (*p* < 0.0001) (Figure 2B). No significant difference was detected in the abundance of FLOT1 and CD81 between the groups (Figure 2B). Additional EV marker proteins were detected by TMT‐MS including the tetraspanins CD9, CD82, TSG101, and ALIX (PDCD6IP). The expression of CD9 was lowest in EVs from Mildly‐deg samples and increased in Deg samples (*p* = 0.0438) (Figure 2C). No significant difference in the abundance of CD82, TSG101, and ALIX was observed between the groups (Figure 2C). As in the Western blot experiments, organelle markers were absent in the TMT‐MS data.

The lipid‐bilayered membrane structures and size distribution of EVs were confirmed by TEM (Figure 2D). In addition, EV size was further assessed using NTA, which showed that regardless of the degeneration degree, the average size (diameter, mean ± SD) of the highest concentration in our samples was 85.2 ± 12.8 nm for exosomes (70–150 nm), 155.9 ± 10.1 nm for intermediate microvesicles (151–500 nm), and 522.6 ± 49.1 nm for large microvesicles (501–1000 nm) Table 3. The EV concentration was also assessed in these three subgroups (Figure 2E). Increasing concentrations of exosomes and intermediate microvesicles was found with progressive IVD degeneration. The exosome concentration increased from 1.19 × 10^5^ ± 1.58 × 10^4^ (Particles/mL)/ng DNA in Non‐deg to 1.97 × 10^5^ ± 1.89 × 10^4^ (Particles/mL)/ng DNA in Mildly‐deg samples (*p* = 0.0375) (Figure 2E(a)). The intermediate microvesicle concentration increased from 3.20 × 10^4^ ± 3.39 × 10^3^ (Particles/mL)/ng DNA in Non‐deg to 5.61 × 10^4^ ± 4.71 × 10^3^ (Particles/mL)/ng DNA in Mildly‐deg samples (*p* = 0.0090) (Figure 2E(b)). The concentration of large microvesicles was not significantly different between the groups (Figure 2E(c)).

**TABLE 3:** EV size information.

	Subgroups	Average diameter (nm)	SD (nm)
Overall size (regardless of the degeneration degree of origin tissue)	Exosome (70–150 nm)	85.2	12.8
	Intermediate microvesicles (151–500 nm)	155.9	10.1
	Large microvesicles (501–1000 nm)	522.6	49.1
Degeneration degree of origin tissue	Subgroups	Average diameter (nm)	SD (nm)
Non‐degenerate	Exosome (70–150 nm)	82.3	15.4
	Intermediate microvesicles (151–‐500 nm)	159.7	15.5
	Large microvesicles (501–1000 nm)	523.8	58.9
Mildly‐degenerate	Exosome (70–150 nm)	84.3	11.7
	Intermediate microvesicles (151–500 nm)	154.7	8.1
	Large microvesicles (501–1000 nm)	527.9	57.0
Degenerate	Exosome (70–150 nm)	89.6	13.8
	Intermediate microvesicles (151–500 nm)	155.4	8.7
	Large microvesicles (501–1000 nm)	523.6	29.4

The findings confirmed the presence of EV markers and detected the lowest expression of ANXA5 and CD9 in samples originating from the Mildly‐deg group. The majority of EVs from IVD cells are exosomes and intermediate microvesicles with an increasing amount from degenerating tissue.

### 3.2 The proteomic profile and differential protein expression in EVs from human IVD cells

**FIGURE 3:**
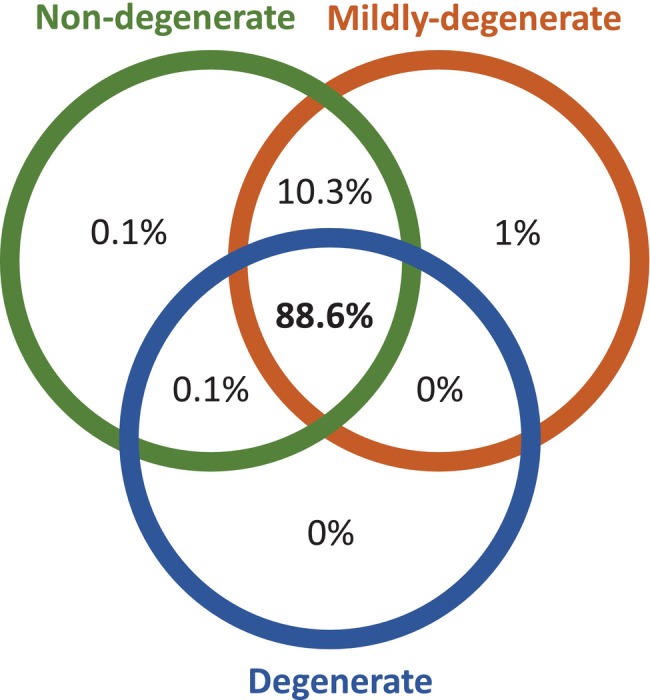
EV protein distribution in the Non‐deg, Mildly‐deg, and Deg samples. Venn diagram representing overlapping and unique proteins in EVs from IVD tissue, green, non‐deg, orange, mildly‐deg, and blue, Deg.

TMT‐MS analysis was performed to establish the proteomic landscape and evaluate protein abundance in cell‐derived EVs from IVD tissue of increasing degree of degeneration. A total of 3427 proteins were identified across the samples and 1121 of them were detected with high FDR confidence (FDR ≤1%). Most of the detected proteins, 993 proteins (88.6% of total proteins), were shared between the groups. 115 proteins (10.3%) were detected only in the Non‐deg and Mildly‐deg groups while 1 protein (FCGBP) (0.1%) was detected only in the Non‐deg and Deg groups. (Figure 3). In addition, 1 protein (0.1%) was exclusively detected in the Non‐deg group (TRIM47) and 11 proteins (1%) were exclusively detected in the Mildly‐deg group (P4HA1, PAFAH1B1, ATP6V1E1, TMBIM1, HLA‐A, EPS8L2, SFTPB, ECPAS, HEXB, SNX3, MAPK14). No protein was shared exclusively between the Mildly‐deg and the Deg groups and no protein was detected exclusively in the Deg group (Figure 3).

### 3.3 Functional annotation and pathway analysis of proteins detected in one or two groups

As mentioned earlier, 115 proteins were detected in the Non‐deg and Mildly‐deg samples and 11 were exclusively detected in the Mildly‐deg samples (Figure 3). A surface antigen CDH2 (CD325) was highly expressed only in the Non‐deg and Mildly‐deg samples, which might serve as a marker to distinguish EVs of such tissue. In addition, more ECM proteins were detected only in the Non‐deg and Mildly‐deg samples: COL2A1, COL28A1, SPARCL1, and SMOC1. Furthermore, a metalloproteinase inhibitor, TIMP1, was also exclusively detected in the Non‐deg and Mildly‐deg samples. These findings indicated that EVs from Non‐deg and Mildly‐deg tissue carry ECM components and molecules promoting ECM production. The cargo, if transported to the extracellular space, close or at a distance, may facilitate ECM generation and/or regeneration.

**FIGURE 4:**
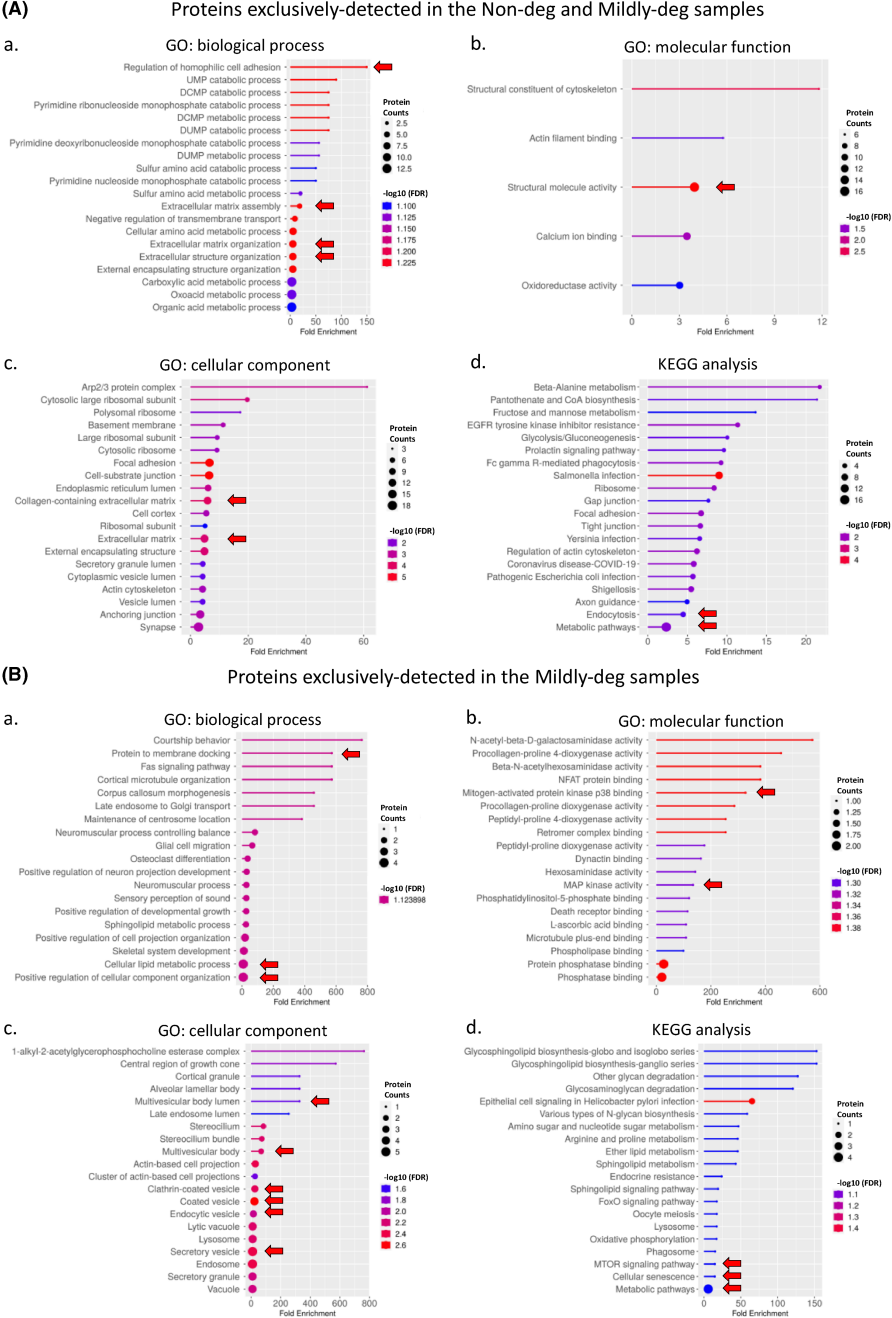
Proteomic cargo annotation and pathways of EV proteins shared by Non‐deg and Mildly‐deg samples and exclusively detected in the Mildly‐deg samples. (A and B) Gene Ontology (GO) within the (a) biological process, (b) molecular function, and (c) cellular component terms and (d) the Kyoto Encyclopedia of Genes and Genomes (KEGG) pathway analyses presenting the top 20 annotations and pathways with strong association with EV cargo proteins detected in the (A) Non‐deg and mildly‐deg samples and exclusively detected in the (B) mildly‐deg samples. GO and KEGG false discovery rate (FDR) cut‐off: 0.1. Annotation/pathway size: minimum 2, maximum 2000. Circle size refers to the protein counts, colours represent the logarithmic scale of the FDR (−Log_10_ (FDR)), and the *x*‐axis describes the fold change of proteins involved in the enriched GO annotations or KEGG pathways. The red triangles highlight the annotations/pathways of interest.

The functional annotations of Gene Ontology (GO) terms related to biological functions of the shared proteins in Non‐deg and Mildly‐deg samples were associated with cell adhesion, ECM, multiple catabolic, and metabolic processes (Figure 4A(a)), the molecular function term indicated an involvement in structural molecule activity (Figure 4A(b)), and the cellular component term indicated an involvement in ECM (Figure 4A(c)). The Kyoto Encyclopedia of Genes and Genomes (KEGG) pathway analysis showed associations with endocytosis and metabolic pathways (Figure 4A(d)). The protein–protein interaction (PPI) networks are depicted in Figure S1.

The GO terms of proteins found exclusively in the Mildly‐deg samples showed associations with biological functions related to membrane docking, cellular lipid metabolic process, and cellular component organization (Figure 4B(a)), the molecular function term indicated an involvement in mitogen‐activated protein kinase p38 binding and MAP kinase activity (Figure 4B(b)), and the cellular component term indicated involvement in multivesicular body and vesicle (Figure 4B(c)). The KEEG pathways with strong associations include the MTOR signaling pathway, cellular senescence, and metabolic pathways (Figure 4B(d)). The PPI network is depicted in Figure S2.

The analyses showed that the functional proteins shared by the Non‐deg and Mildly‐deg groups were highly related to catabolic and metabolic processes, and so were the functional proteins detected exclusively in the Mildly‐deg group.

### 3.4 Functional annotation and pathway analysis of proteins detected in all groups

Although expressed in all groups, there was a significant difference in the relative abundance of shared proteins. Comparing the top 20 expressed proteins in each group illustrates this difference (Table S1). Thirteen proteins (25%) were found only in the top 20 list of the Non‐deg group (CILP, PCOLCE, MAN1A1, LTBP2, ECM2, FBLN1, HAPLN1, FARP1, ITIH5, NQO1, BGN, HAL, and ANXA1). Thirteen proteins (25%) were found only in the top 20 list of the Mildly‐deg group (FSTL1, HEL‐S‐51, APOH, BRD9, EFEMP1, C1S, AHSG, C1R, AFM, DCN, APOA1, PPARD, and CTSD). Eighteen proteins (34.6%) were found only in the top 20 list of the Deg group (PTX3, CD107a (LAMP1), SCARB2, ITIH2, TMPRSS13, TTYH3, CD107b (LAMP2), ATP1B3, CD276, SLC1A5, ABI3BP, CD26 (DPP4), CD13 (ANPEP), H4, CD105 (ENG), KRT6A, ACAN, and KRT14). Six proteins (11.5%) were shared between the Non‐deg and Mildly‐deg groups (SPARC, NUCB1, CLEC3B, SERPING1, EIF5A, and CHI3L1), 1 protein (1.9%) was shared between the Mildly‐deg and Deg groups (LTF), and 1 protein (1.9%) was shared between the Non‐deg and Deg groups (NTN1). No protein was shared by all three groups in the top 20 list.

**FIGURE 5:**
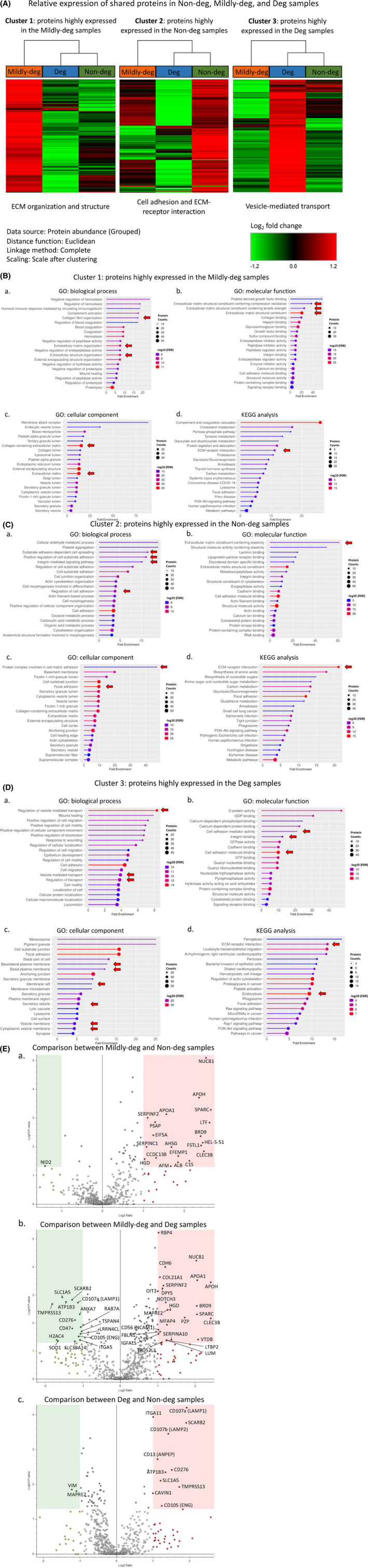
Comparison and categorization of the proteomic profile of EVs derived from cells of non‐, mildly‐, and degenerate human IVD tissue. (A) Heatmap of differentially expressed proteins (DEPs), the heatmap is divided into three clusters presented side by side. The red‐green colour strip represents differential Log_2_ fold change: enrichment/high expression was depicted in red and depletion/low expression in green, and the horizontal line represents each DEP. The column colour represents the IVD tissue degeneration: orange, mildly‐deg; green, non‐deg; and blue, deg. *n* = 3–6. (B–D) GO within the (a) biological process, (b) molecular function, and (c) cellular component terms and (d) the KEGG pathway analyses presenting the top 20 annotations and pathways with strong association of shared DEPs in (B) mildly‐deg, (C) non‐deg, and (D) deg samples. GO and KEGG FDR cut‐off: 0.05. Annotation/pathway size: minimum 2, maximum 2000. Circle size refers to the protein counts, colours represent the logarithmic scale of the FDR (−Log_10_ (FDR)), and the *x*‐axis describes the fold change of DEPs involved in the enriched GO annotations or KEGG pathways. The red arrows highlight the annotations/pathways of high interest. (E) Volcano plots showing the abundance comparisons of DEPs in the (a) mildly‐deg vs. non‐deg, (b) mildly‐deg vs. deg, and (**c**) deg vs. non‐deg groups. The *x*‐axis shows the Log_2_ fold change, and the *y*‐axis shows the negative Log_10_ of the *p*‐value (a larger number represents higher significance). The horizontal cut‐off shows the significance threshold *p* ≤ 0.05 and the vertical cut‐off shows the Log_2_ fold change ≥ 1. Dark‐coloured dots are EV proteins that are significant within the threshold. Dark red and dark green colours represent upregulated and downregulated DEPs, respectively. The grey dots are below the significance threshold. *n* = 3–6.

A heatmap was generated based on the differential protein expression, the heatmap was split into 3 parts, from top to bottom and presented side by in Figure 5A. The Mildly‐deg group showed the largest overall difference of the 3 clusters.

We then performed unbiased functional analyses to determine the functions, processes, and pathways that the enriched proteins were involved in. The shared proteins of Cluster 1: proteins highly expressed in the Mildly‐deg samples were associated with the GO biological function terms of collagen fibril organization and ECM and extracellular structure organization (Figure 5B(a)). The molecular function term indicated an involvement in ECM structural constituents (Figure 5B(b)). The cellular component term indicated an involvement in the ECM (Figure 5B(c)). KEGG pathway analysis indicated an association with ECM–receptor interaction (Figure 5B(d)). The GO enrichment analysis of Cluster 2: proteins highly expressed in the Non‐deg samples showed an association with the biological function terms of cell adhesion and integrin‐mediated signaling (Figure 5C(a)). The molecular function term indicated that the shared proteins were associated with ECM constituents conferring elasticity (Figure 5C(b)). The cellular component terms indicated that the shared proteins were associated with protein complexes involved in cell–matrix adhesion and focal adhesion (Figure 5C(c)). KEGG pathway analysis indicated an association with ECM–receptor interaction (Figure 5C(d)). The shared proteins of Cluster 3: proteins highly expressed in the Deg samples were associated with the biological function term of vesicle‐mediated transport (Figure 5D(a)). The molecular function term indicated an involvement in cell adhesion mediator activity and cell adhesion molecule/integrin binding (Figure 5D(b)). The cellular component term indicated an involvement in the plasma and vesicle membrane (Figure 5D(c)). KEGG pathway analysis indicated an association with ECM–receptor interaction and endocytosis (Figure 5D(d)).

Based on the differentially expressed protein (DEP) abundance and the protein clustering pattern, we explored the DEP hierarchical interactions. The PPI networks of the top 10% ranked proteins in each cluster were identified using the cytoHubba plugin with the maximum clique centrality (MCC) ranking method, determining the number and size of maximal cliques the hub protein is part of. The PPI networks evaluate the top 3 hub proteins of each cluster (Figure S3). Cluster 1: COL1A1, DCN, and POSTN (Figure S3A); Cluster 2: HSPA4, TCP1, and RPS3 (Figure S3B); and Cluster 3: ITGB1 (CD29), CD44, and RHOA (Figure S3C).

Our findings suggest that EVs derived from IVD cells from tissue with different degrees of degeneration share most cargo proteins, while the relative expression of the shared proteins varies. Together, the analyses indicated that the 3 clusters were related to (1) cell adhesion and ECM–receptor interaction, which were detected with high abundance in the Non‐deg samples; (2) ECM organization and structure, which were detected with high abundance in the Mildly‐deg samples; and (3) vesicle‐mediated transport, which were detected with high abundance in the Deg samples.

We generated volcano plots to evaluate the fold difference between two groups. We included only proteins detected at low FDR in all 3 groups. Comparing the Mildy‐deg to the Non‐deg samples, we found 20 proteins that were significantly higher, and the abundance of 1 protein was significantly lower in the Mildly‐deg samples (Table S2‐1). SPARC (Log_2_ fold change (FC) = 2.65, −Log_10_ *p* = 3.29) and NID2 (Log_2_ FC = −1.40, −Log_10_ *p* = 1.33) were the most and least abundant proteins (Figure 5E(a)). Comparing the Mildly‐deg to the Deg samples showed that the abundance of 25 proteins was significantly higher, and the abundance of 16 proteins was significantly lower in the Mildly‐deg group (Table S2‐2). APOH (Log_2_ FC = 2.42, −Log_10_ *p* = 3.46) and TMPRSS13 (Log_2_ FC = −1.81, −Log_10_ *p* = 2.68) were the most and least abundant proteins (Figure 5E(b)). Nine (25%) upregulated proteins (SERPINF2, SPARC, CLEC3B, BRD9, HEL‐S‐51, APOA1, APOH, and HGDNUCB1) were shared between the comparisons of Mildly‐deg vs. Non‐deg and the Mildly‐deg vs. Deg samples. While no overlap was found in the proteins with lower expression. The functional annotations of GO terms and KEGG pathway analyses related to the proteins detected significantly higher or lower levels are depicted in Figure S4A–C.

The difference in relative abundance of DEPs was less pronounced comparing EVs from cells of Deg to Non‐deg IVD tissue. The abundance of only 11 proteins was significantly higher in the Deg samples (Table S2‐3). The abundance of two proteins was significantly lower (Table S2‐3). CD107a (LAMP1) (Log_2_ FC = 1.92, −Log_10_ *p* = 4.21) and VIM (Log_2_ FC = ‐1.22, −Log_10_ *p* = 1.85) were the most and least abundant proteins (Figure 5E(c)). GO functional annotation and KEGG pathway analyses comparing DEPs in the Deg to Non‐deg samples are depicted in Figure S4D.

These findings indicate that EV cargo from the Mildly‐deg samples is the most distinct. The upregulated proteins were strongly associated with ECM and EV. The downregulated proteins were associated with transport, ECM–receptor interaction, protein digestion and absorption, and necroptosis (Figure S4).

### 3.5 The relative abundance of selected protein groups

**FIGURE 6:**
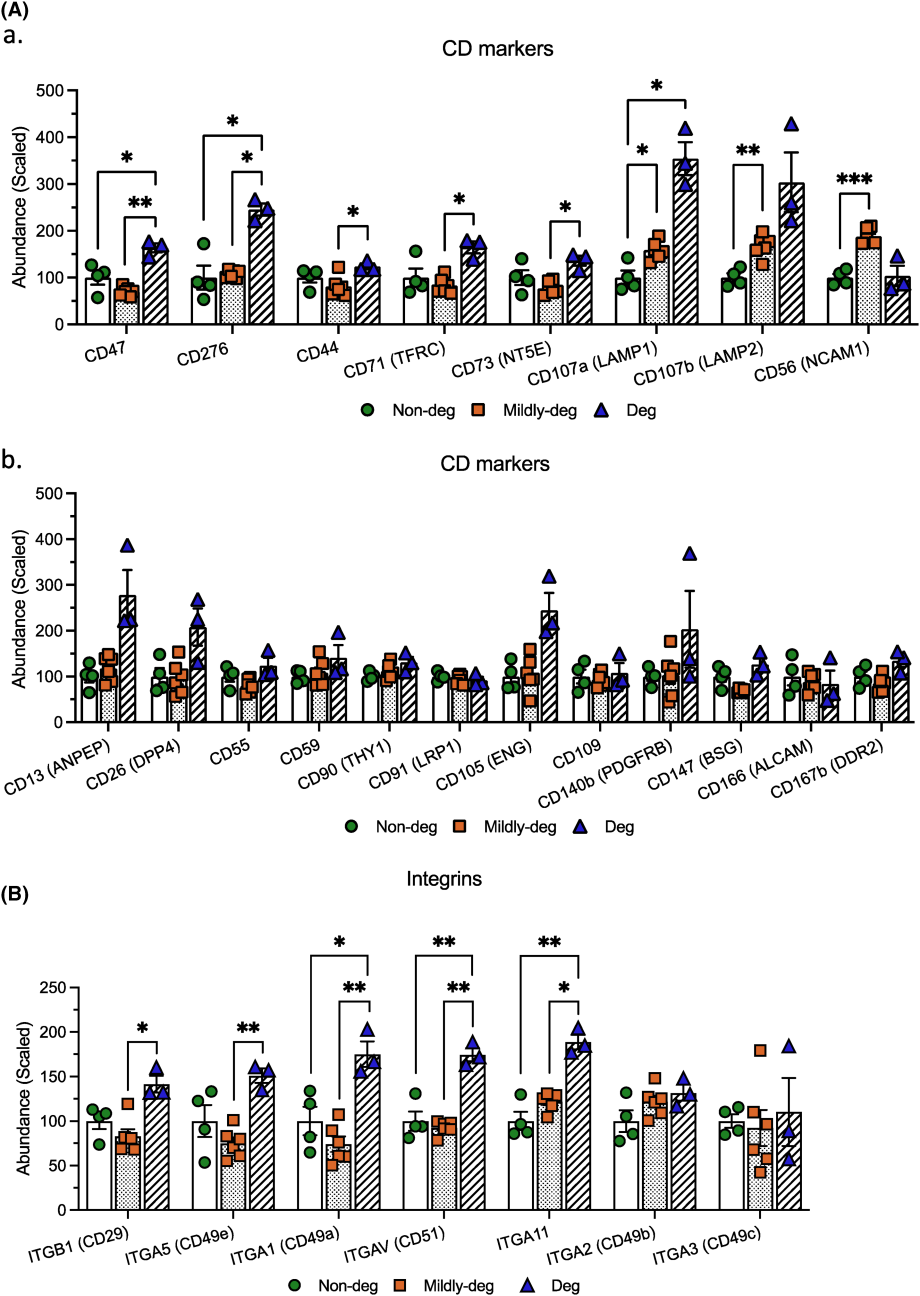
Comparison of selected shared DEPs: Cell surface markers. TMT‐MS analyses depicting the relative abundance of the DEPs in the (A) Cluster of differentiation (CD) markers and (B) Integrins. *n* = 3–6. Values are presented as mean ± SEM. *, **, and *** indicate statistically significant changes assessed by Dunnett's T3 multiple comparisons under Welch's ANOVA tests: *p* < 0.05, *p* < 0.01, and *p* < 0.001.

**FIGURE 7:**
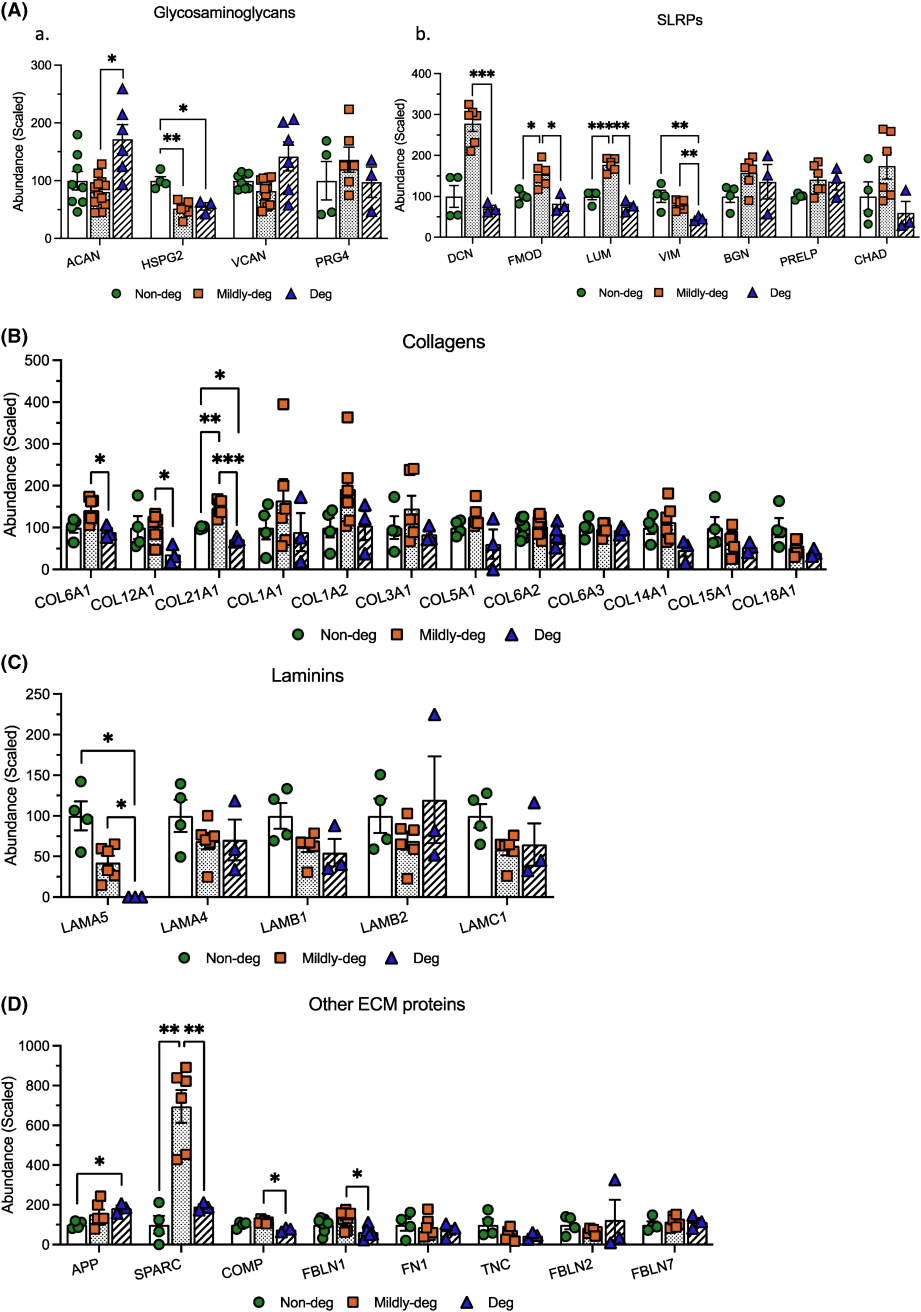
Comparison of selected shared DEPs: ECM proteins. TMT‐MS analyses depicting the relative abundance of the DEPs in the ECM component. (A). (a) Glycosaminoglycans and (b) Small leucine‐rich proteoglycans (SLRPs). (B) Collagens. C. Laminins. D. Other ECM proteins. *n* = 3‐12. Values are presented as mean ± SEM. *, **, and *** indicate statistically significant changes assessed by Dunnett's T3 multiple comparisons under Welch's ANOVA tests: *p* < 0.05, *p* < 0.01, and *p* < 0.001.

**FIGURE 8:**
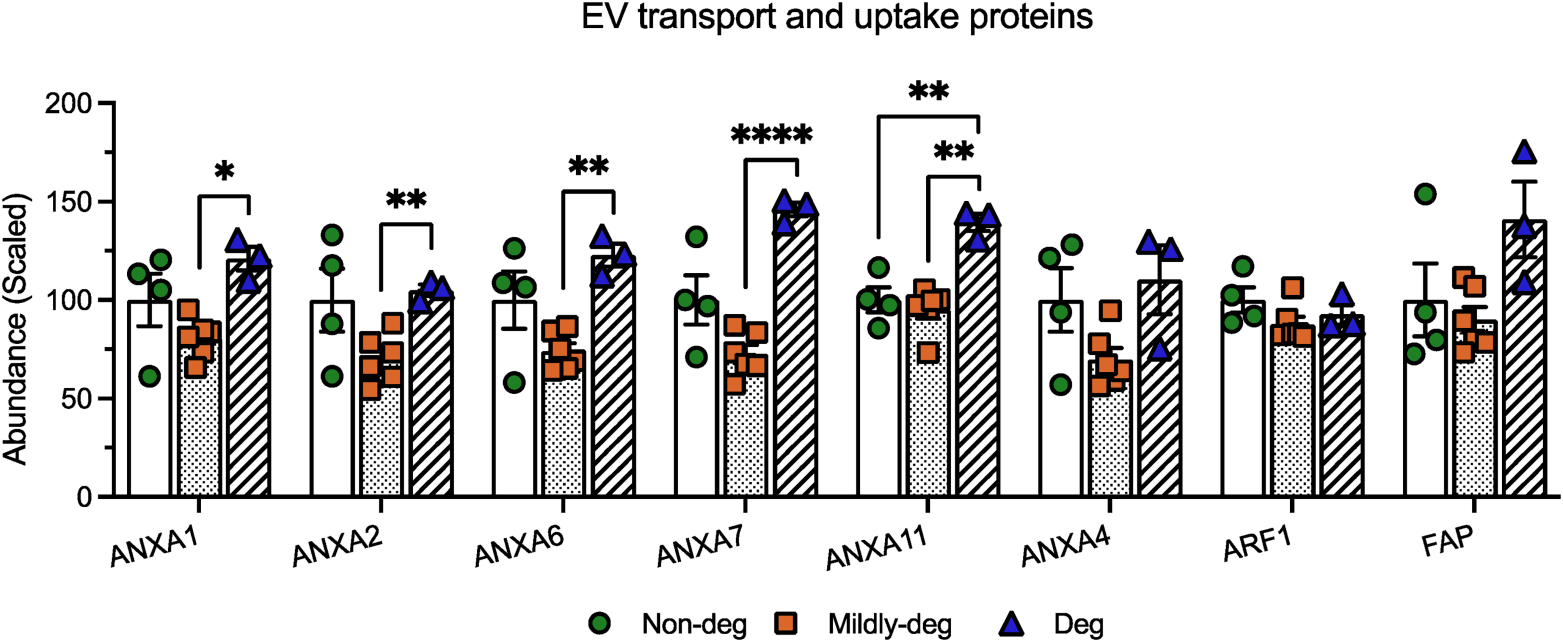
Comparison of selected shared DEPs: Proteins involved in EV transport and uptake. TMT‐MS analyses depicting the relative abundance of the DEPs involved in EV transport and uptake. *n* = 3–6. Values are presented as mean ± SEM. *, **, and **** indicate statistically significant changes assessed by Dunnett's T3 multiple comparisons under Welch's ANOVA tests: *p* < 0.05, *p* < 0.01, and *p* < 0.0001.

**FIGURE 9:**
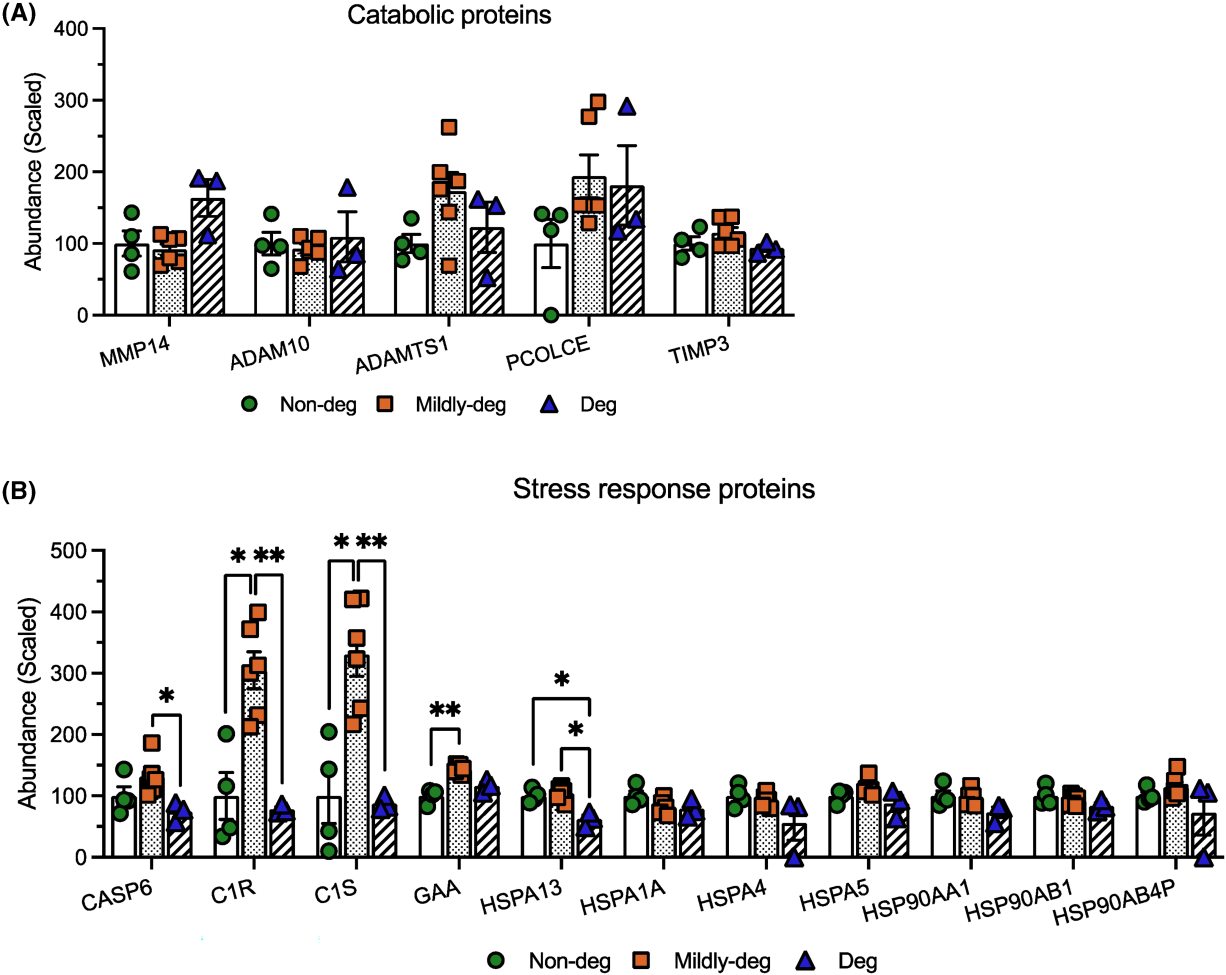
Comparison of selected shared DEPs: proteins involved in catabolic activity and stress response. TMT‐MS analyses depicting the relative abundance of the DEPs involved in (A) catabolic activity and (B) stress response. *n* = 3–6. Values are presented as mean ± SEM. * and ** indicate statistically significant changes assessed by Dunnett's T3 multiple comparisons under Welch's ANOVA tests: *p* < 0.05 and *p* < 0.01.

To further interpret the pattern of shared cargo, selected DEPs were categorized into 4 domains: Cell surface markers (Figure 6), ECM components (Figure 7), proteins involved in EV transport and uptake (Figure 8), and proteins involved in catabolic activity and stress response (Figure 9).

Relative abundance of 7 out of 8 detected cell surface markers increased with degeneration and had the highest expression in the Deg group (scaled abundance) CD47 (*p* = 0.0424 and *p* = 0.0071), CD276 (*p* = 0.0179 and *p* = 0.0195), CD44 (*p* = 0.0156), CD71 (TFRC) (*p* = 0.0262), CD73 (NT5E) (*p* = 0.0372), CD107a (LAMP1) (*p* = 0.0475 and *p* = 0.0162), and CD107b (LAMP2) (*p* = 0.0028). While the relative abundance of CD56 (NCAM1) was highest in the Mildly‐deg group (*p* = 0.0002) (Figure 6A(a)). No significant difference was detected in the abundance of CD13 (ANPEP), CD26 (DPP4), CD55, CD59, CD90 (THY1), CD91 (LRP1), CD105 (ENG), CD109, CD140b (PDGFRB), CD147 (BSG), CD166 (ALCAM), and CD167b (DDR2) (Figure 6A(b)). The cell surface markers CD10 (Neprilysin), CD46, CD99, CD140a (PDGFRA), and CD151 were detected in all groups but the abundance was too low to calculate the relative expression.

The abundance of integrins ITGB1 (CD29), ITGA5 (CD49e), and ITGA1 (CD49a) fluctuated. ITGB1 (*p* = 0.0126) and ITGA5 (*p* = 0.0021) were significantly higher in Deg compared to Mildly‐deg EVs. ITGA1 (*p* = 0.0448 and *p* = 0.0096), ITGAV (CD51) (*p* = 0.0062 and *p* = 0.0050), and ITGA11 (*p* = 0.0029 and *p* = 0.0122) increased progressively with degeneration. No significant difference was found in the abundance of ITGA2 (CD49b) and ITGA3 (CD49c). (Figure 6B). In addition, ITGB3 (CD61) and ITGB5 were detected in all groups, but the abundance was too low to calculate the relative expression.

The abundance of ECM proteins was also variable. In the glycosaminoglycan group, ACAN (core protein and isoform 3) showed the highest abundance in the Deg group and was significantly higher compared to the Mildly‐deg group (*p* = 0.0342). HSPG2 was significantly lower in EVs from Mildly‐deg (*p* = 0.0048) and Deg (*p* = 0.0147) groups compared to the Non‐deg group. (Figure 7A(a)).

The abundance of the small leucine‐rich proteoglycan (SLRP) DCN was the highest in the Mildly‐deg group with a significant difference compared to the Deg group (*p* = 0.0003). FMOD and LUM were significantly more abundant in EVs from Mildy‐deg compared to the Non‐deg (*p* = 0.0151 and *p* = 0.0007) and Deg (*p* = 0.0250 and *p* = 0.0016) groups. The abundance of VIM was the lowest in the Deg samples with a significantly higher abundance in the Non‐deg (*p* = 0.0069) and Mildy‐deg (*p* = 0.0029) groups. No significant difference was detected in the abundance of BGN, PRELP, and CHAD between the groups. (Figure 7A(b)).

Numerous collagens were detected in the EVs. COL6A1, COL12A1, and COL21A1 were the lowest in the Deg group, which was significant between the Mildly‐deg and Deg groups for COL6A1 (*p* = 0.0235) and COL12A1 (*p* = 0.0284) and between all groups for COL21A1 (*p* = 0.0045, *p* = 0.0131, and *p* = 0.0001). No significant difference was detected in the abundance of COL1A1, COL1A2, COL3A1, COL5A1, COL6A2 (core protein and isoform 2C2A), COL6A3, COL14A1, COL15A1, and COL18A1. (Figure 7B). In addition, COL4A1, COL5A2, and COL11A1 were detected in all groups, but the abundance was too low to calculate the relative expression.

Laminins detected include LAMA5, which was significantly lower in the Deg group (*p* = 0.0267 and *p* = 0.0106), whereas no difference in abundance was found in LAMA4, LAMB1, LAMB2, and LAMC1 between the groups (Figure 7C). In addition, LAMA2 was detected in all groups, but the abundance was too low to calculate the relative expression.

Some other ECM protein abundance also showed a significant difference. Amyloid‐beta precursor protein (APP) abundance was significantly higher in the Deg compared to the Non‐deg groups (*p* = 0.0192). SPARC abundance was significantly higher in the Mildy‐deg compared to Non‐deg and Deg groups (*p* = 0.0011 and *p* = 0.0049). COMP (*p* = 0.0123) and FBLN1 (core protein and isoform C) (*p* = 0.0111) were significantly higher in the Mildly‐deg compared to the Deg groups. (Figure 7D).

The results confirm that proteins shared between the groups are present at a variable abundance, which might have implications for cell‐to‐cell communication.

In the group of proteins involved in EV transport and uptake, numerous Annexins were detected. The expression of ANXA1, ANXA2, ANXA6, and ANXA7 was the lowest in the Mildly‐deg group with significantly higher abundance in the Deg group (*p* = 0.0135, *p* = 0.0015, *p* = 0.0058, and *p* < 0.0001). ANXA11 was significantly higher in the Deg compared to the Non‐deg (*p* = 0.0097) and Mildly‐deg (*p* = 0.0012) groups. No significant difference was detected in the abundance of ANXA4, ARF1, and FAP (Figure 8).

These findings suggest increased cellular activities involved in EV transport and uptake, as well as apoptosis in EVs from degenerate tissue.

Proteins involved in catabolic activities and stress responses include MMP14, ADAM10, ADAMTS1, PCOLCE, and TIMP3; however, no significant difference was detected in their abundance (Figure 9A).

DEPs involved in apoptotic and inflammatory processes and heat shock proteins were detected in the stress response proteins domain. The expression of CASP6, C1R, C1S, and GAA was highest in the Mildly‐deg group, C1R and C1S were significantly lower in the Non‐deg (*p* = 0.0154 and *p* = 0.0188) and Deg (*p* = 0.0019 and *p* = 0.0030) groups, GAA was significantly lower in the Non‐deg (*p* = 0.0064) group and CASP6 was significantly lower in the Deg (*p* = 0.0193) group. The expression of HSPA13 was the lowest in the Deg group, compared to the Non‐deg (*p* = 0.0296) and Mildly‐deg (*p* = 0.0172) groups. No significant difference was detected in the abundance of HSPA1A, HSPA4, HSPA5, HSP90AA1, HSP90AB1, and HSP90AB4P (Figure 9B).

These findings showed that stress response proteins were the highest in the Mildly‐deg group, suggesting increased cellular activities involved in apoptosis and inflammation.

## 4 DISCUSSION

Although previous studies have investigated the secretory profile of NP cells^52^ and MSCs exposed to IVD cell conditioned media,^53^ no prior investigation has compared the proteomic profiles of EV cargo from human IVD cells derived from non‐degenerate, mildly‐degenerate, and degenerate tissue while using the same culturing and purification conditions. Our findings revealed that most EV proteins were shared between the groups while a minor proportion were uniquely identified in one or two groups, potentially representing new molecular indicators of IVD health. Moreover, our results show that the EV protein clusters are associated with specific features depending on the level of IVD degeneration. This provides a framework for understanding fundamental IVD biology from an EV perspective and may propose candidate biomarkers for IVD disease.

### 4.1 EV characteristics

The size of IVD cell‐derived EVs has previously been investigated. A study by Lu et al. reported a 30–100 nm range of exosomes derived from human degenerating NP tissue,^1^ while Zhao et al. and Feng et al. reported that exosomes derived from human degenerating NP tissue had an average diameter of 120 and 126 nm, respectively, at the peak concentration.^54^, ^55^ Song et al. suggest that exosomes are produced at an elevated level from human NP cells of tissue with high degenerative grade. The average size of NP exosomes in Song's study was 147 and 157 nm from non‐degenerating and degenerating tissue, respectively.^56^ Ambrosio et al. reported that exosomes from healthy NP and NP^Tie2+^ tissue were in the 106 nm range.^36^ In addition, Sun et al. isolated exosomes from AF cell CM from non‐degenerating and degenerating human tissue and found that the size of the AF exosomes of both groups was in the 50–150 nm range with no significant difference.^57^ It is unclear if Sun et al. combined cells from the inner and outer AF or if the outer AF was excluded. We excluded outer AF tissue but combined cells from NP and inner AF tissue, as it is often impossible to determine the difference in surgical tissue samples. Our data is in line with Song et al.^56^ and shows the enrichment of distinct EV populations related to tissue health, demonstrating increased particle quantity with higher degeneration of exosomes and intermediate microvesicles. In addition, the exosome size determined in our samples is in line with the reports of Lu et al. and Sun et al.^1^, ^57^ The overall average size (mean ± SD) of the highest concentration in our samples, regardless of the degeneration degree of the tissue, was 85.2 ± 12.8 nm for exosomes, 155.9 ± 10.1 nm for intermediate microvesicles, and 522.6 ± 49.1 nm for large microvesicles.

### 4.2 EV marker profile

The marker profile of EVs derived from human IVD cells has not been systematically determined. Previous studies evaluating EVs from IVD cells have used 5 markers to validate their purification protocol^1^, ^36^, ^54^, ^55^, ^56^, ^57^ (Table 4). Each study used 2–3 out of the 5 markers. CD63 was used in all studies, TSG101 and CD9 in 5 and 4 out of the 6 studies, and CD81 and ALIX were only used 1 time each. We detected all markers except CD63 in our samples. Widening our scope to other connective tissues, 37 exosome markers have been described and served as references for developing and validating a predictive model for the diagnosis of transient ischemic attack^58^ (Table 4). We detected 8 of the 37 exosome markers, 3 of which (CD29, CD44, and CD105) are general MSC EV markers.^59^, ^60^

**TABLE 4:** Comparison of EV markers detected in our study with that of previous studies.

EV marker or family	*MISEV2023* ^12^ suggested	MSC studies^59^, ^60^	TIA study^58^	NP & AF published in^1^, ^36^, ^54^, ^55^, ^56^, ^57^	Our study
TSG101	✔	✔		✔	X c
ALIX (PDCD6IP)	✔			✔	X c
FLOT1/2	✔				X c
Annexins (ANX**)^a^					X
CD1c			✔		
CD2			✔		
CD3			✔		
CD4			✔		
CD8			✔		
CD9	✔	✔	✔	✔	X c
CD10		✔			X c
CD11c			✔		
CD13 (ANPEP)		✔			X c
CD14		Negative marker	✔		
CD19			✔		
CD20			✔		
CD24			✔		
CD25			✔		
CD26 (DPP4)					X
CD29 (ITGB1)		✔	✔		X c
CD31			✔		
CD34		Negative marker			
CD40			✔		
CD41b			✔		
CD42a			✔		
CD44		✔	✔		X c
CD45		Negative marker	✔		
CD46					X
CD47	✔				X c
CD55					X
CD56 (NCAM1)			✔		X c
CD59	✔				X c
CD62P			✔		
CD63	✔	✔	✔	✔	
CD69			✔		
CD71					X
CD73 (NT5E)	✔	✔			X c
CD81	✔	✔	✔	✔	X c
CD82	✔				X c
CD86			✔		X c
CD90 (THY1)		✔			X c
CD91 (LRP1)					X
CD99					X
CD105 (ENG)		✔	✔		X c
CD107a/b (LAMP1/2)	✔				X c
CD109					X
CD133/1			✔		
CD140a/b					X
CD142			✔		
CD146			✔		
CD147 (EMMPRIN (BSG))	✔				X c
CD151					X
CD166 (ALCAM)					X
CD167b (DDR2)					X
CD209			✔		
CD276					X
CD325 (CDH2)					X
CD326			✔		
HLA‐ABC			✔		
HLA‐DRDPDQ			✔		
MCSP			✔		
ROR1			✔		
SSEA‐4			✔		
Heterotrimeric G proteins (GNA*)^a^	✔				X c
Integrins (ITGA*/ITGB*)	✔		✔ ITGA5 (CD49e)		X c
Transferrin receptor (TFRC)	✔				X c
Heparan sulphate proteoglycans including syndecans (SDC*)	✔				X c
ADAM10	✔				X c
Glypicans (GPC1)	✔				X c
Caveolins (CAV*)	✔				X c
Syntenin	✔				X c (SDCBP)
HSC70 (HSPA8)	✔				X c
HSP84 (HSP90AB1)	✔				X c
Actin (ACT*)	✔				X c
Tubulin (TUB*)	✔				X c
Enzymes (GAPDH)	✔				X c
VPS4A/B	✔				
ARRDC1	✔				
Apolipoproteins (APO*)	✔				X c
Immunoglobulins (IG*)	✔				X c
Albumin	✔				X c
YWAH* (14‐3‐3*)	✔				X c
AGO*	✔				X c
HSP90AA/B	✔				X c
TGFBI	✔				X c
HSPA13	✔				X c
LDHA/B	✔				X c
Tamm‐Horsfall protein (Uromodulin/UMOD; urine)	✔				
Histones (HIST1H**)	Subtypes of EVs and/or pathologic/atypical state, and/or novel separation method				X
Lamin A (LMNA)					X
GRP94 (HSP90B1)					X
BIP (HSPA5)					X
Actinin1/4 (ACTN1/4)					X
Lamin C (LMNC)					
VDAC, cytochrome C (CYC1)					
TOM20					
Calnexin (CANX)					
GM130 (GOLGA2)					
Autophagosomes: LC3 (MAP1LC3A)					
Complement	✔				X c
Fibrinogen	✔				X c
PDGFD	✔				X c
Interleukins	✔				X c (IL34)
Fibronectin	✔				X c (FN1)
Collagens (COL**)	✔				X c
MFGE8	✔				X c
Galectin3‐binding protein	✔				X c (LGALS3BP)
Fetuin‐A (AHSG)	✔				X c
TGFB1/2	✔				
IFNG	✔				
VEGFA	✔				
FGF1/2	✔				
EGF	✔				
CD5L	✔				

*Note*: “✔” indicates the proteins reported in published studies. “X c” indicates proteins the detected in our study which were also reported in published studies. “X” indicates the proteins detected in our study.

Abbreviation: TIA, transient ischemic attacks.

^a^ XX** or XX* is used to indicate families of multiple proteins.

We also compared our determined EV marker profile to identified MSC EV markers,^59^, ^60^ and the EV markers suggested in *Minimal information for studies of extracellular vesicles* (MISEV2023)^12^ (Table 4). We detected all 7 positive markers (CD10, CD13, CD29, CD44, CD73, CD90, and CD105) and none of the negative MSC EV markers described by Kim et al.^59^ and Maumus et al.^60^ Compared to the *MISVE2023* guidelines, we could detect 47 of the 63 potential markers and marker families in EVs from IVD cells independent of degeneration level (Table 4). Of note, we did not detect the commonly reported EV marker CD63. We also did not detect the accessory proteins VPS4A/B and ARRDC1, cytokines, and growth factors, except IL‐34 and CD5L (Table 4). The absence of CD63 in our samples may be because it was below the detection limit. Or it may be due to the culture or purification conditions we used. We used the low‐particulate, xeno‐free, protein‐free collection media for EVs, supplemented with 1% dFBS, which was different from the abovementioned studies. Song et al. and Sun et al. applied 10% dFBS in their studies,^56^, ^57^ while other researchers did not specify the concentration of dFBS but reported the use of exosome‐free media.^1^, ^36^, ^54^, ^55^

The detected EV markers were presented across samples of different degeneration levels. Based on our data, we propose that the surface antigens CD276, CD13 (ANPEP), CD26 (DPP4), CD55, CD91 (LRP1), CD109, CD140a/b, CD166 (ALCAM), CD167b (DDR2), CD10, CD46, CD99, and CD151 can be considered for the detection of EVs derived from human IVD cells. In addition, CDH2 (CD325) may be used to segregate EVs of non‐degenerate and mildly‐degenerate IVD tissue from those of degenerate IVD tissue, as it was only detected in the non‐ and mildly‐degenerate samples.

### 4.3 EV cargo

No previous study to our knowledge has evaluated the difference in protein cargo in EVs from human IVD tissue of different degrees of degeneration. Some studies, however, have evaluated circular RNA profiles. Song et al. found an elevated expression of NP cell circular RNA_0000253 and microRNA‐141‐5p in degenerating samples compared to those in the non‐degenerating samples.^56^ Sun et al. reported that AF exosomes from degenerating tissue exerted a pro‐vascularization effect by the expression of inflammatory mediators that promoted cell migration. On the other hand, the exosomes derived from the non‐degenerating AF tissue prevented blood vessel ingrowth and retained the IVD homeostasis. Together, their findings demonstrated that AF exosomes derived from IVD tissue with different conditions acted oppositely to each other in the degradation process of IVD.^57^ These findings may suggest that EV cargo and function vary depending on and reflect the pathophysiological state of the IVD tissue that the parental cells arose from. Another factor that affects EV cargo is the culturing condition. Previous studies demonstrated that EV quantity and composition are modulated in MSCs, prostate cancer cells, and neuroblastoma cells by the type of culture media and culture dish/chamber/bioreactor, and the supplements added to the culture media (e.g., serum‐free condition).^61^, ^62^, ^63^, ^64^ Our data demonstrate that the degeneration level of IVD tissue is reflected in the EV protein cargo, indicating distinct EV populations with specific biological functions and/or of a distinct nature related to tissue health. However, most proteins, 88.6%, were present in all samples, although, at different expression levels. Only 10.3% were exclusively detected in the non‐ and mildly‐degenerate samples and 1% was detected only in the mildly‐degenerate samples. The shared proteins are associated with (1) cell adhesion and ECM–receptor interaction, (2) ECM organization and structure, and (3) vesicle‐mediated transport in the non‐, mildly‐, and degenerate samples, respectively.

### 4.4 Cargo related to cell adhesion

EVs are known to carry integrins as cargo and in the surrounding membrane.^65^, ^66^, ^67^ Integrins are transmembrane adhesion receptors composed of an α and a β chain with crucial roles in cell–matrix interaction and signal transduction affecting survival proliferation and migration.^68^, ^69^, ^70^ Distinct sets of integrin receptors were detected in the IVD cells.^68^, ^71^, ^72^, ^73^ We detected some of them in IVD cell EVs. ITGB1 (CD29), ITGA5 (CD49e), ITGA1 (CD49a), ITGAV (CD51), ITGA11, ITGA2 (CD49b), and ITGA3 (CD49c) were detected in high abundance and ITGB3 (CD61) and ITGB5 were detected in low abundance in all samples. Alpha subunits interacting with ITGB1, ITGB3, and ITGB5 were also detected, including ITGA1 (CD49a), ITGA2 (CD49b), ITGA3 (CD49c), ITGA5 (CD49e), ITGAV (CD51), and ITGA11. α5β1, αvβ1, αvβ3, and αvβ5 are fibronectin‐binding integrins, α1β1, α2β1, and α11β1 are collagen‐binding integrins, and α3β1 is a laminin binding integrin.^71^, ^72^, ^73^, ^74^ The role of integrin receptors in the context of EVs is not well described in the IVD research field. Some studies suggested that integrin composition is involved in EV docking and uptake^65^, ^66^ and can direct EVs for uptake in distinct cell types or tissues.^67^ Hoshino et al. demonstrated that exosomal integrins were associated with lung and liver metastasis and could be used in organ‐specific metastasis prediction.^67^ In the context of IVD health, integrin‐mediated cell adhesion has been shown to exhibit protective properties. Wu et al. demonstrated that NP cell survival was promoted through the activation of a fibronectin/ITGB1/syndecan‐4 complex and the focal adhesion kinase (FAK)/phosphoinositide 3‐kinase (PI3K)/AKT pathway.^75^ In addition, integrin activation protects AF cells by attenuating cell apoptosis through the upregulation of the extracellular signal‐regulated kinase (ERK) pathway.^76^ Integrins are also instrumental in matrix organization and could potentially support tissue organization and regeneration.

### 4.5 Cargo related to ECM organization and structure

Numerous ECM components were detected in IVD cell‐derived EVs. Collagens are major ECM components of connective tissues and interact with macromolecules such as SLRPs.^77^ Non‐degenerate NP and inner AF tissue have a high abundance of type II collagen,^74^ and Type II collagen was exclusively detected in our non‐ and mildly‐ degenerate samples. In addition, the abundance of other collagens, such as COL6A1, COL12A1, and COL21A1, and some SLRPs, such as DCN, FMOD, and LUM, were also found at a higher abundance in EVs from the mildly‐degenerate IVD tissue. Aggrecan is another major component with an important functional role in IVD tissue homeostasis. In contrast to collagen, the abundance of aggrecan and CD44 was significantly higher in the EVs from degenerated tissue. Vimentin (VIM), a type III intermediate filament protein, plays a fundamental role in cell mechanics^78^ and tissue damage and repair.^79^ We detected a significantly decreased VIM expression in our EVs of the degenerate samples, which is in line with our previous finding, demonstrating a lower *VIM* expression in human IVD cells from degenerate tissue at the transcriptomic and proteomic levels.^80^ Others have shown that VIM is absent in IVDs of patients with degenerative disc disease^81^ and it has been suggested to play a role in the development of tissue fibrosis.^82^ SPARC (secreted protein acidic and rich in cysteine) is a calcium‐binding glycoprotein matrix protein.^83^, ^84^ It is one of the most abundant non‐collagenous proteins in non‐mineralized tissues. It has been reported that SPARC expression decreased with increasing age and disc degeneration in human.^85^, ^86^ In addition, SPARC‐null mice exhibit age‐dependent disc degeneration, low back pain, and elevated levels of inflammatory mediators in the IVDs.^87^, ^88^, ^89^, ^90^ Our results showed that SPARC abundance was exceptionally high in EVs from the mildly‐degenerate IVD tissue. In addition, SPARCL1 and SMOC1 were detected exclusively in the non‐ and mildly‐degenerate samples. Our data show a differential distribution of ECM‐associated molecules related to the degree of degeneration the tissue originated from. The high abundance of molecules with regenerative effects detected in the mildly‐degenerate samples could indicate a greater potential to increase ECM synthesis in recipient cells. However, further functional investigations are required to validate this.

### 4.6 Cargo related to EV transport and uptake

Seven annexins related to EV transport and uptake were identified in this study. Annexins (lipocortins) are calcium‐dependent phospholipid‐ and membrane‐binding proteins, classified into 5 families (A–E). The annexin A family is commonly detected in humans and contains 12 members (annexin A1–11 and A13).^84^ Annexins are involved in numerous cellular processes, and they have anti‐inflammatory properties. They regulate cell growth and apoptosis and can influence EV synthesis, transport, and uptake.^91^, ^92^ For example, Annexin A1 showed anti‐inflammatory effects in rheumatoid arthritis by regulating glucocorticoids.^93^ Annexin A6 regulated autophagy and endocytic trafficking processes in hepatocytes.^94^ The increased expression of the annexins in EVs from degenerating IVD tissue may reflect an attempt to reduce inflammatory mediators. It may also indicate an increased EV synthesis and transport in degenerating tissue, in line with the increasing trend revealed by the nanoparticle tracking results.

### 4.7 Cargo related to catabolic activity and stress response

EVs are known to carry cargo related to catabolic activity and stress response. We detected 18 proteins in this category. ADAMs (A Disintegrin and Metalloprotease) are transmembrane proteases, which mediate ectodomain shedding and play important roles in cell communication, cytokine secretion, and transmembrane signaling.^95^, ^96^ ADAM10 is among the most abundant proteins identified in EVs and has been suggested as a novel marker for small EVs.^97^, ^98^ ADAMTS (ADAM with ThromboSpondin motifs) are secreted metalloendopeptidases, with roles in tissue morphogenesis and pathophysiological remodeling.^99^ The heat shock proteins respond to temperature, environmental, physiological, and clinical stress and demonstrate cellular protection against apoptosis during radiotherapy, chemotherapy, and immunotherapy.^100^ Lischnig et al. demonstrated that ADAM10 and ADAMTS1 were enriched in small EVs, whereas a majority of the heat shock proteins were enriched in large EVs from breast cancer cell lines.^96^ We also detected these proteases in our dataset, however, without a differential expression. This indicates that the proteins detected in this category are not associated with the degenerative degree of the original tissue in IVD cell‐derived EVs.

### 4.8 Study limitations

The isolation and expansion of cells will affect cell phenotype and may also select for more proliferative cell populations, including progenitor cells, which are known to reside within IVD tissue.^17^, ^18^, ^19^, ^20^ Cell expansion may thus affect EV cargo and its functional properties compared to EVs found in vivo or collected from explant cultures. A problem with explant cultures is the variable cell numbers present in degenerate and non‐degenerate tissue along with the effect of aging on cellularity. This could be addressed by using 3D cultures but the effects of different 3D systems, oxygen and glucose levels, and loading conditions are currently not well established. To minimize these effects, we used primary cells at passage 1, cultured under a constant glucose level of 2.25 g/L, and EV‐depleted serum during the collection phase. Future studies should evaluate the effect of culturing conditions on IVD cell‐derived EV cargo and determine conditions yielding EVs with the highest regenerative potential.

## 5 SUMMARY AND CONCLUSION

EV cargo can affect numerous cellular processes, including cell differentiation, cell senescence, and modulate immune and inflammatory responses. A better understanding of how IVD‐cell‐derived EVs and their cargo change in the degeneration process may provide information related to the molecular mechanisms underlying IVD degeneration and new treatment modalities for IVD‐related low back pain.

## AUTHOR CONTRIBUTIONS

*Conceptualization*: L. L., H. A., and L. H. *Methodology*: L. L. and H. A. *Validation*: L. L., A. S., H. C., and L. H. *Formal analysis*: L. L. *Investigation*: L. L. *Resources*: M. G., R. B., J. O., P. J., and L. H. *Data curation*: L. L. and A. S. *Writing—original draft preparation*: L. L. *Writing—review and editing*: L. L., H. A., A. S., M. G., R. B., J. O., P. J., H. C., and L. H. *Visualization*: L. L. *Supervision*: H. C. and L. H. *Project administration*: L. H. *Funding acquisition*: L. H. All authors have read and agreed to the published version of the article.

## CONFLICT OF INTEREST STATEMENT

The authors declare no conflicts of interest.

## Supporting information


**Data S1:** Supporting Information

## Data Availability

All data generated or analyzed during this study are included in the manuscript and supporting files. The raw data and materials used to support the findings of this study are available from the corresponding author upon request.
