# Supplementary material for: The proteomic landscape of extracellular vesicles derived from human intervertebral disc cells
Source: JOR Spine. 2024 Nov 5;7(4):e70007. doi: 10.1002/jsp2.70007 (PMC11538033; doi:10.1002/jsp2.70007)
Supplement: Supplementary file 1 — Data S1: Supporting Information [file JSP2-7-e70007-s001.pdf]

Supplementary materials

The proteomic landscape of extracellular vesicles derived from human intervertebral disc cells

Li Li[1,3,4], Hadil Al-Jallad[2], Aiwei Sun[5], Miltiadis Georgiopoulos[1,3], Rakan Bokhari[1,3,6], Jean Ouellet[1,2,3], Peter Jarzem[1,3], Hosni Cherif[1,3,4], Lisbet Haglund[1,2,3,4 *]

[1]Department of Surgery, Division of Orthopaedics, McGill University, Montreal, Quebec, Canada,
[2]Shriners Hospital for Children, Montreal, Quebec, Canada,
[3]The McGill Scoliosis and Spine Group, McGill University Health Centre, Montreal, Quebec, Canada,
[4]The Orthopaedic Research Laboratory, Research Institute of the McGill University Health Centre, Montreal, Quebec, Canada,
[5]Department of Anatomy and Cell Biology, McGill University, Montreal, Quebec, Canada.
[6]Department of Surgery, Division of Neurosurgery, Faculty of Medicine, King Abdulaziz University, Jeddah, Saudi Arabia.

Summary

Supplemental tables:

Table S1. The top 20 enriched proteins in EVs of cells from Non-deg, Mildly-deg, and Deg IVD tissue.
Table S2-1. Upregulated and downregulated EV proteins, comparing Mildly-deg vs. Non-deg samples.
Table S2-2. Upregulated and downregulated EV proteins, comparing Mildly-deg vs. Deg samples.
Table S2-3. Upregulated and downregulated EV proteins, comparing Deg vs. Non-deg samples.

Supplemental figures:

Figure S1. PPI network of shared EV proteins in the Non-deg and Mildly-deg samples.
Figure S2. PPI network of EV proteins exclusively detected in the Mildly-deg samples.
Figure S3. PPI network of shared cargo proteins in the Non-deg, Mildly-deg, and Deg IVD samples.
Figure S4. Proteomic cargo annotation and pathway analysis of upregulated and downregulated proteins of Mildly-deg vs. Non-deg, Mildly-deg vs. Deg, and Deg vs. Non-deg groups.

Table S1. The top 20 enriched proteins in EVs of cells from Non-deg, Mildly-deg, and Deg IVD tissue.

Abundance rank (Descending)	Non-deg		Mildly-deg		Deg	
	Protein accession	Gene Symbol	Protein accession	Gene Symbol	Protein accession	Gene Symbol
1	O75339	CILP	P09486	SPARC	P26022	PTX3
2	P63241-1	EIF5A	Q02818	NUCB1	O95631	NTN1
3	P05155	SERPING1	P05452	CLEC3B	P11279	CD107a (LAMP1)
4	Q15113	PCOLCE	P02788	LTF	Q14108-1	SCARB2
5	Q02818	NUCB1	Q12841	FSTL1	P19823	ITIH2
6	P33908	MAN1A1	P02774	HEL-S-51	Q9BYE2	TMPRSS13
7	Q14767	LTBP2	P05155	SERPING1	Q9C0H2-1	TTYH3
8	P36222	CHI3L1	P02749	APOH	P13473-1	CD107b (LAMP2)
9	P09486	SPARC	Q9H8M2-1	BRD9	P54709	ATP1B3
10	O94769	ECM2	P63241-1	EIF5A	Q5ZPR3	CD276
11	P05452	CLEC3B	Q12805	EFEMP1	Q15758-1	SLC1A5
12	O95631	NTN1	P09871	C1S	P02788	LTF
13	P23142-4	FBLN1	P02765	AHSG	Q7Z7G0	ABI3BP
14	P10915	HAPLN1	P00736	C1R	P27487	CD26 (DPP4)
15	Q9Y4F1	FARP1	P43652	AFM	P15144	CD13 (ANPEP)
16	Q86UX2-1	ITIH5	P07585	DCN	P62805	H4
17	P15559-1	NQO1	P02647	APOA1	P17813	CD105 (ENG)
18	P21810	BGN	Q03181	PPARD	P02538	KRT6A
19	P42357	HAL	P07339	CTSD	P16112	ACAN
20	P04083	ANXA1	P36222	CHI3L1	P02533	KRT14

Table S1 presents the top 20 enriched proteins with the highest abundance in EVs of cells from non-degenerate, mildly-degenerate, and degenerate IVD tissue.

Table S2-1. Upregulated and downregulated EV proteins, comparing Mildly-deg vs. Non-deg samples.

	Upregulated proteins			Downregulated proteins		
Abundance rank (Descending)	Protein accession	Gene symbol	Description	Protein accession	Gene symbol	Description
1	P09486	SPARC	Sparc	Q14112-1	NID2	Nidogen-2
2	Q02818	NUCB1	Nucleobindin-1			
3	P05452	CLEC3B	Tetranectin			
4	P02788	LTF	Lactotransferrin			
5	Q12841	FSTL1	Follistatin-related protein 1			
6	P02774	HEL-S-51	vitamin D-binding protein			
7	P02749	APOH	Beta-2-glycoprotein 1			
8	Q9H8M2-1	BRD9	Isoform 5 of Bromodomain-containing protein 9			
9	P63241-1	EIF5A	Eukaryotic translation initiation factor 5A-1			
10	P02768-1	ALB	Serum albumin			
11	Q12805	EFEMP1	EGF-containing fibulin-like extracellular matrix protein 1			
12	P09871	C1S	Complement C1s subcomponent			
13	P02765	AHSG	Alpha-2-HS-glycoprotein			
14	P43652	AFM	Afamin			
15	P02647	APOA1	Apolipoprotein A-I			
16	P07602-1	PSAP	Prosaposin			
17	Q96M89-1	CCDC138	Coiled-coil domain-containing protein 138			
18	P08697-1	SERPINF2	Alpha-2-antiplasmin			
19	Q93099	HGD	Homogentisate 1,2-dioxygenase			
20	P01008	SERPINC1	Antithrombin-III			

Table S2-1 lists the upregulated and downregulated EV proteins from the comparison between mildly-degenerate and non-degenerate samples.

Table S2-2. Upregulated and downregulated EV proteins, comparing Mildly-deg vs. Deg samples.

	Upregulated proteins			Downregulated proteins		
Abundance rank (Descending)	Protein accession	Gene symbol	Description	Protein accession	Gene symbol	Description
1	P09486	SPARC	Sparc	P11279	CD107a (LAMP1)	Lysosome-associated membrane glycoprotein 1
2	Q02818	NUCB1	Nucleobindin-1	Q14108-1	SCARB2	Lysosome membrane protein 2
3	P05452	CLEC3B	Tetranectin	Q5ZPR3	CD276	CD276 antigen
4	P02774	HEL-S-51	Vitamin D-binding protein	P17813	ENG	Endoglin
5	P02749	APOH	Beta-2-glycoprotein 1	P54709	ATP1B3	Sodium/potassium-transporting ATPase subunit beta-3
6	Q9H8M2-1	BRD9	Isoform 5 of Bromodomain-containing protein 9	P51149	RAB7A	Ras-related protein Rab-7a
7	P02647	APOA1	Apolipoprotein A-I	Q8ND94	LRRN4CL	LRRN4 C-terminal-like protein
8	P08697-1	SERPINF2	Alpha-2-antiplasmin	Q15043	SLC39A14	Zinc transporter ZIP14
9	Q14767	LTBP2	Latent-transforming growth factor beta-binding protein 2	Q15758-1	SLC1A5	Neutral amino acid transporter B(0)
10	Q93099	HGD	Homogentisate 1,2-dioxygenase	Q9BYE2	TMPRSS13	Transmembrane protease serine 13
11	P20742	PZP	Pregnancy zone protein	P08648	ITGA5	Integrin alpha-5
12	Q9UK55	SERPINA10	Protein Z-dependent protease inhibitor	P20073-1	ANXA7	Annexin A7
13	P51884	LUM	Lumican	Q08722	CD47	Leukocyte surface antigen CD47

14	P13591-1	NCAM1	Isoform 2 of Neural cell adhesion molecule 1	O14817	TSPAN4	Tetraspanin-4
15	Q14117	DPYS	dihydropyri midinase	P04908	H2A	Histone H2A type 1-B/E
16	P02753	RBP4	Retinol-binding protein 4	P00441	SOD1	Superoxide dismutase [Cu-Zn]
17	Q16890-1	TPD52L1	Tumor protein D53			
18	P23142	FBLN1	Fibulin-1			
19	Q96P44	COL21A1	Collagen alpha-1 (XXI) chain			
20	P55285-1	CDH6	Cadherin-6			
21	Q8WWZ8-1	OIT3	Oncoprotein-induced transcript 3 protein			
22	P55083	MFAP4	Microfibril-associated glycoprotein 4			
23	Q9UM47	NOTCH3	Neurogenic locus notch homolog protein 3			
24	P35858	IGFALS	Insulin-like growth factor-binding protein complex acid labile subunit			
25	Q15555	MAPRE2	Microtubule-associated protein RP/EB family member 2			

Table S2-2 lists the upregulated and downregulated EV proteins from the comparison between mildly-degenerate and degenerate samples.

Table S2-3. Upregulated and downregulated EV proteins, comparing Deg vs. Non-deg samples.

	Upregulated proteins			Downregulated proteins		
Abundance rank (Descending)	Protein accession	Gene symbol	Description	Protein accession	Gene symbol	Description
1	P11279	CD107a (LAMP1)	Lysosome-associated membrane glycoprotein 1	Q15555	MAPRE2	Microtubule-associated protein RP/EB family member 2
2	Q14108-1	SCARB2	Lysosome membrane protein 2	P08670	VIM	Vimentin
3	Q9BYE2	TMPRSS13	Transmembrane protease serine 13			
4	P13473-1	CD107b (LAMP2)	Lysosome-associated membrane glycoprotein 2			
5	P54709	ATP1B3	Sodium/potassium-transporting ATPase subunit beta-3			
6	Q5ZPR3	CD276	CD276 antigen			
7	Q15758-1	SLC1A5	Neutral amino acid transporter B(0)			
8	P15144	ANPEP	aminopeptidase N			
9	P17813	ENG	Endoglin			
10	Q6NZI2-1	CAVIN1	Caveolae-associated protein 1			
11	Q9UKX5	ITGA11	Integrin alpha-11			

Table S2-3 lists the upregulated and downregulated EV proteins from the comparison between degenerate and non-degenerate samples.

PPI network of shared proteins in Non- and Mildly-deg groups

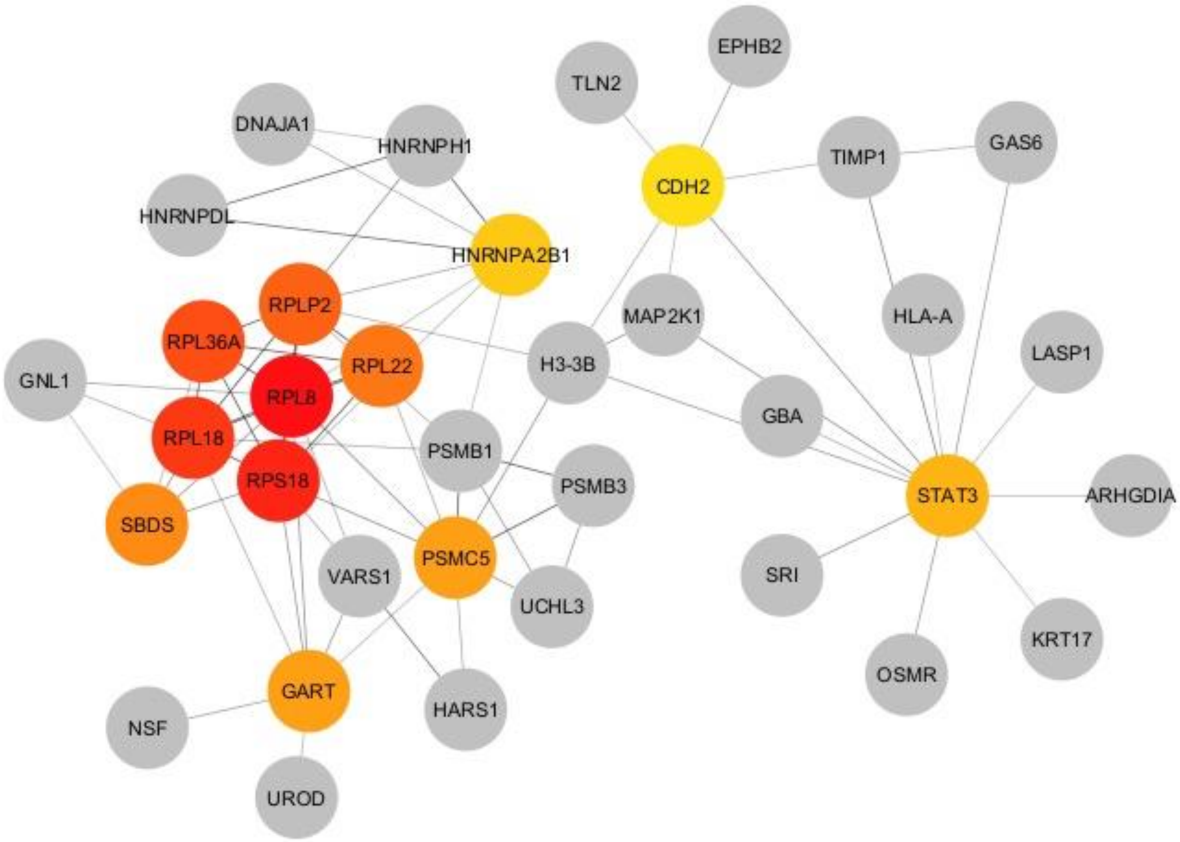

Figure S1. PPI network of shared EV proteins in the Non-deg and Mildly-deg samples. The protein-protein interaction (PPI) network was created using the cytoHubba plugin in Cytoscape with the maximum clique centrality (MCC) ranking method applied to the quantify of the top 10% proteins. Red to yellow colours represent descending ranks and the hierarchical degrees of the hub proteins, indicating a decreasing interaction of them.

The protein-protein interaction (PPI) network of shared extracellular vesicle (EV) proteins in Non-deg and Mildly-deg samples indicated the top 12 hub proteins: RPL8, RPS18, RPL18, RPL36A, RPLP2, RPL22, SBDS, PSMC5, GART, STAT3, HNRNPA2B1, and CDH2 (CD325). Seven of them are ribosomal proteins and proteases, and one is a surface antigen. (**Figure S1**).

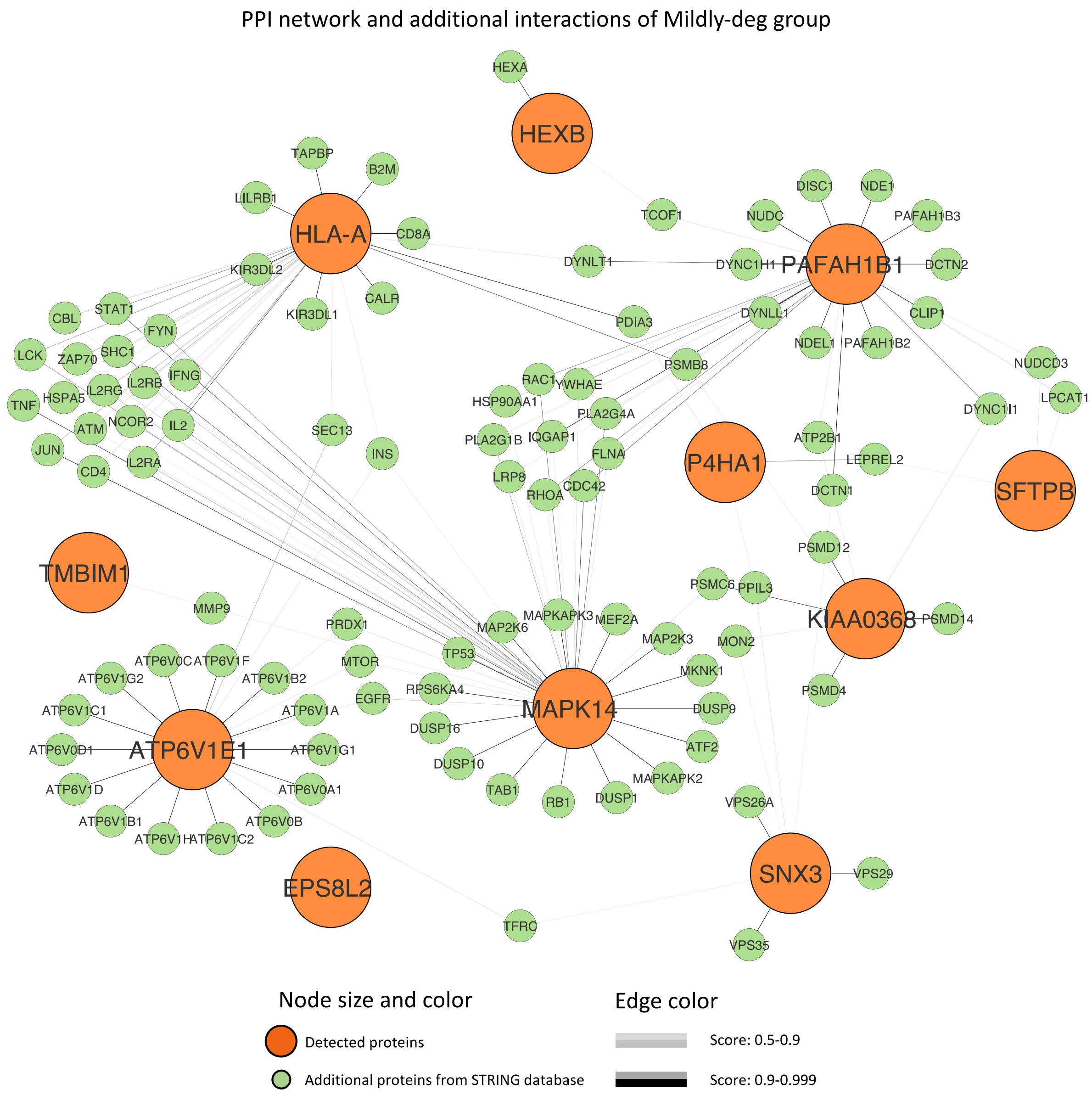

Figure S2. PPI network of EV proteins exclusively detected in the Mildly-deg samples. The PPI network containing proteins detected exclusively in the EVs from cells from Mildly-deg IVD tissue (orange nodes) and the suggested connection proteins (green nodes) from the String database.

SNX3, a protein strongly associated with EV sorting and transport, was detected exclusively in the Mildly-deg group, as well as MAPK14, a mitogen-activated protein kinase involved in extracellular matrix (ECM) remodelling (**Figure S2**). These findings suggested that EVs from IVD tissue of Mildly-deg state transport might undergo in the Mildly-deg state and such EVs transport ECM remodelling molecules.

The PPI network showed that MAPK14 was highly associated with many proteins suggested by the String database, which may serve as the hub protein in the extended network (**Figure S2**).

A. Cluster 1: PPI network of proteins highly expressed in the Mildly-deg group

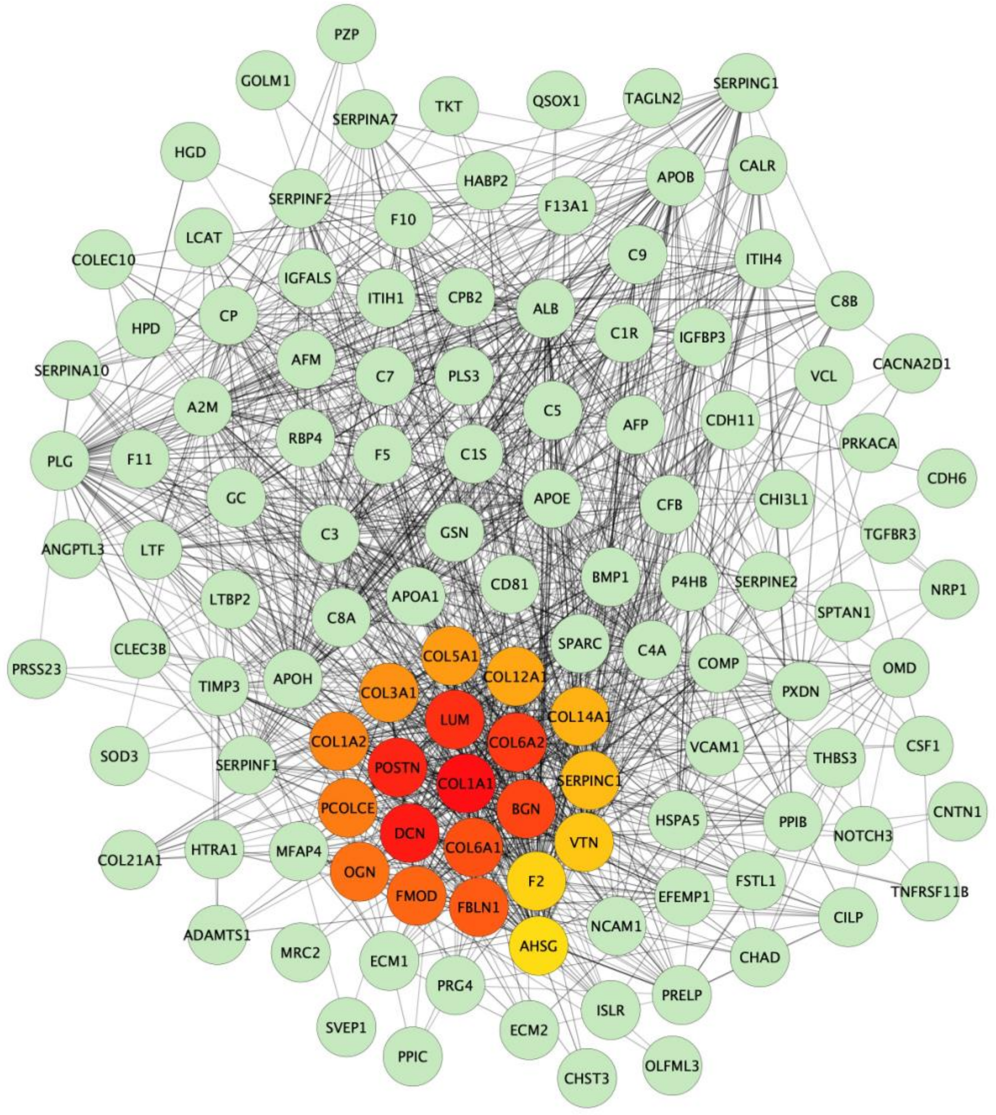

B. Cluster 2: PPI network of proteins highly expressed in the Non-deg group

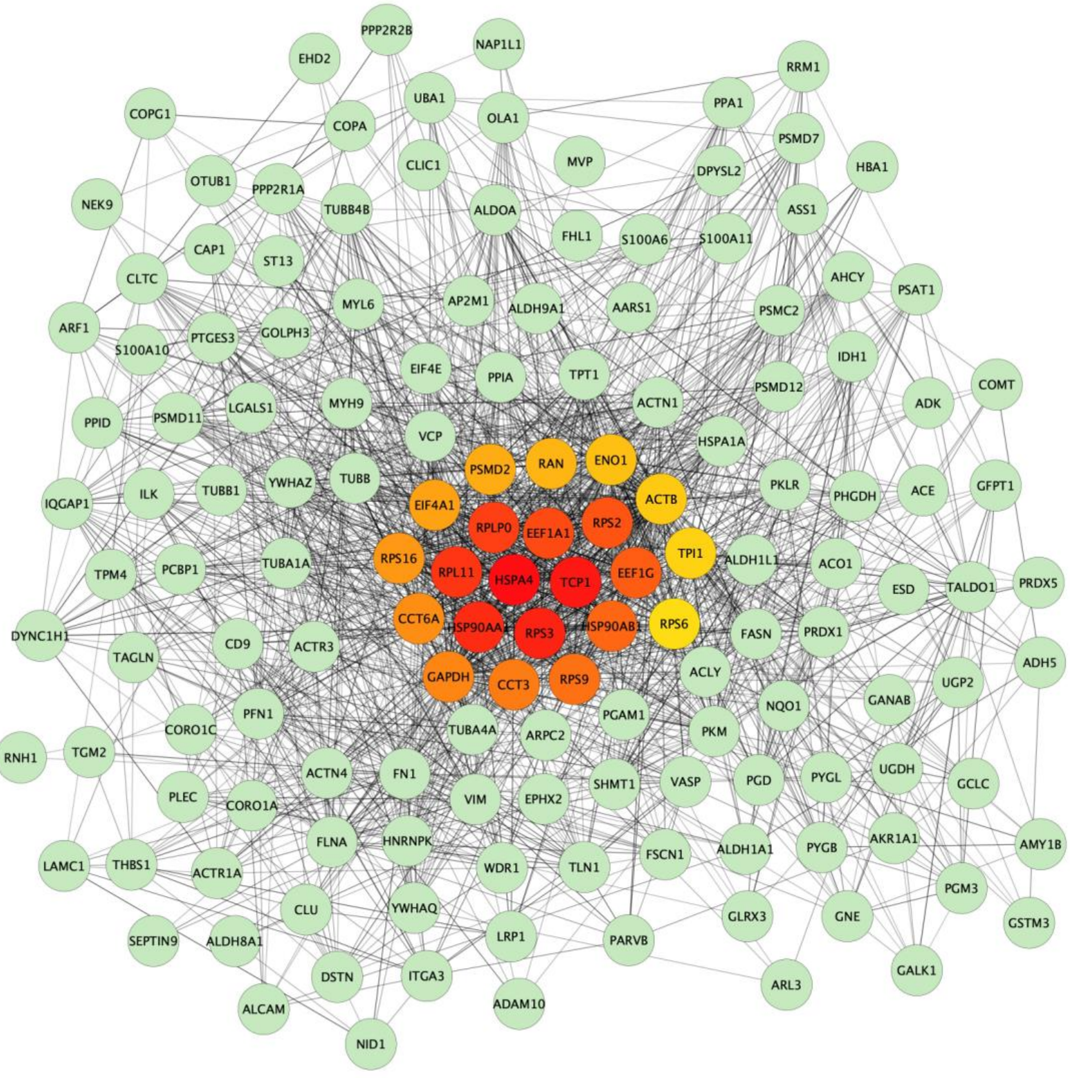

C. Cluster 3: PPI network of proteins highly expressed in the Deg group

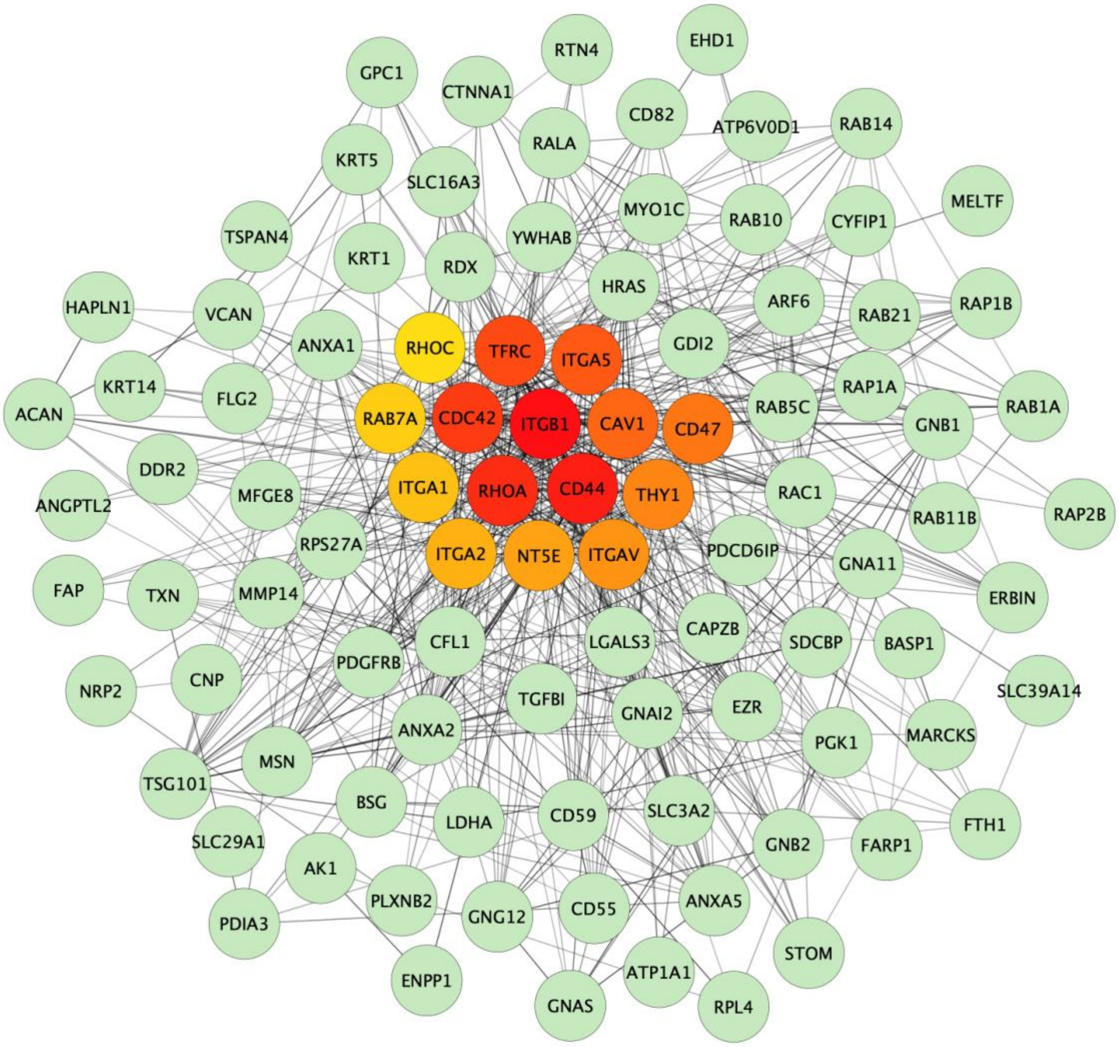

Figure S3. PPI network of shared cargo proteins in the Non-deg, Mildly-deg, and Deg samples. The PPI networks of the proteins highly expressed in the (**A**) Mildly-deg, (**B**) Non-deg, and (**C**) Deg samples. PPI networks were created using the cytoHubba plugin in Cytoscape with the MCC ranking method applied to the quantify of 10% proteins of each cluster. Red to yellow colours represent descending ranks and the hierarchical degrees of the hub proteins, indicating a decreasing interaction of them.

A. Upregulated proteins in the comparison between Mildly-deg and Non-deg samples

a. GO: biological process

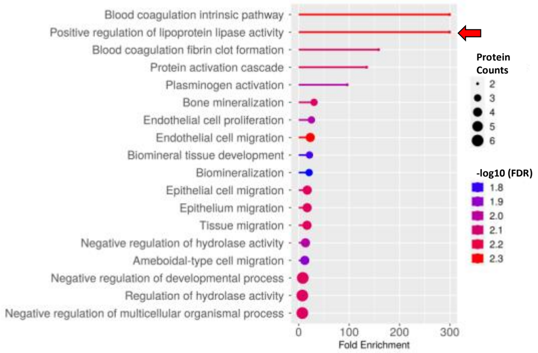

b. GO: molecular function

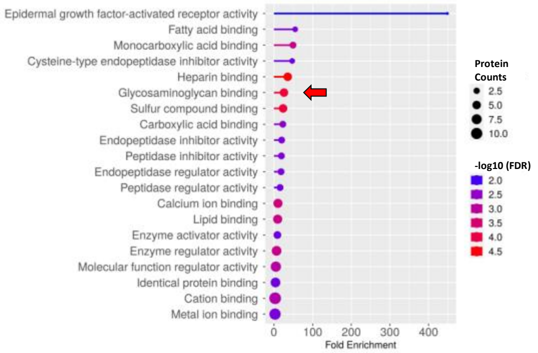

c. GO: cellular component

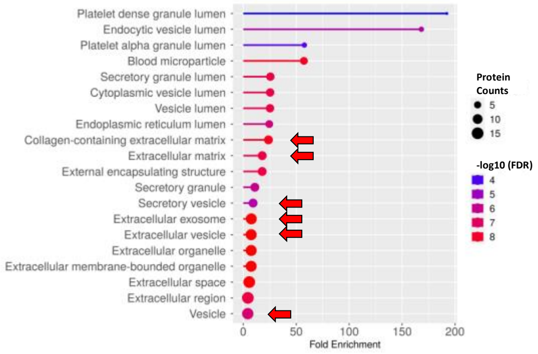

d. KEGG analysis

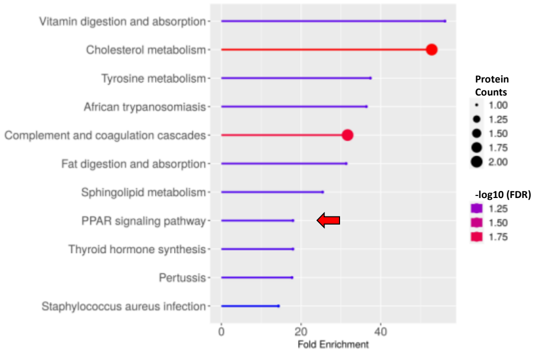

B. Upregulated proteins in the comparison between Mildly-deg and Deg samples

a. GO: biological process

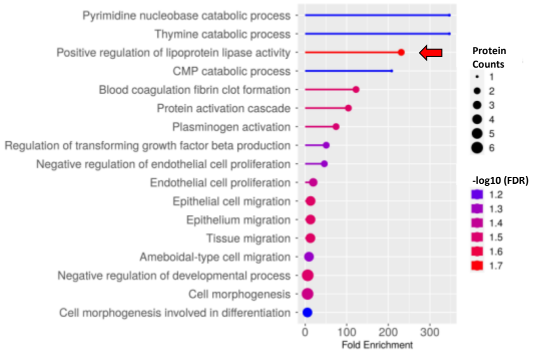

b. GO: molecular function

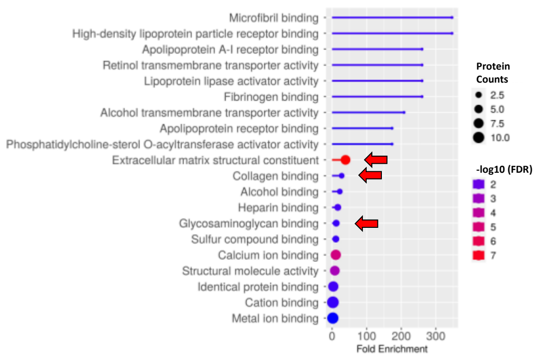

c. GO: cellular component

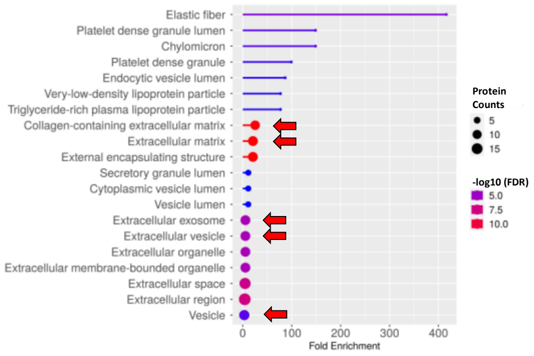

d. KEGG analysis

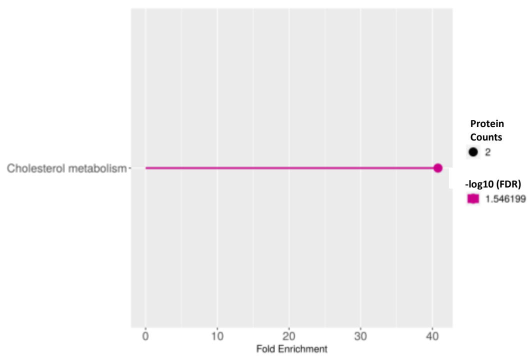

C. Downregulated proteins in the comparison between Mildly-deg and Deg samples

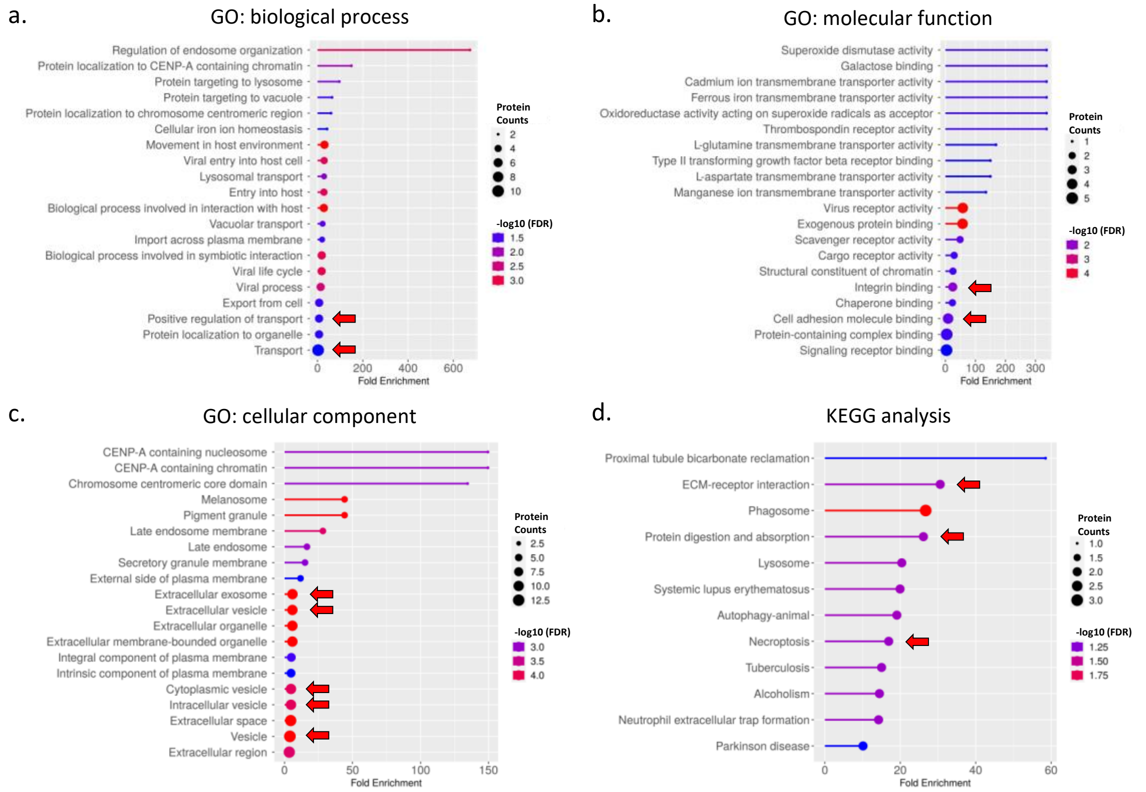

D. Upregulated proteins in the comparison between Deg and Non-deg samples

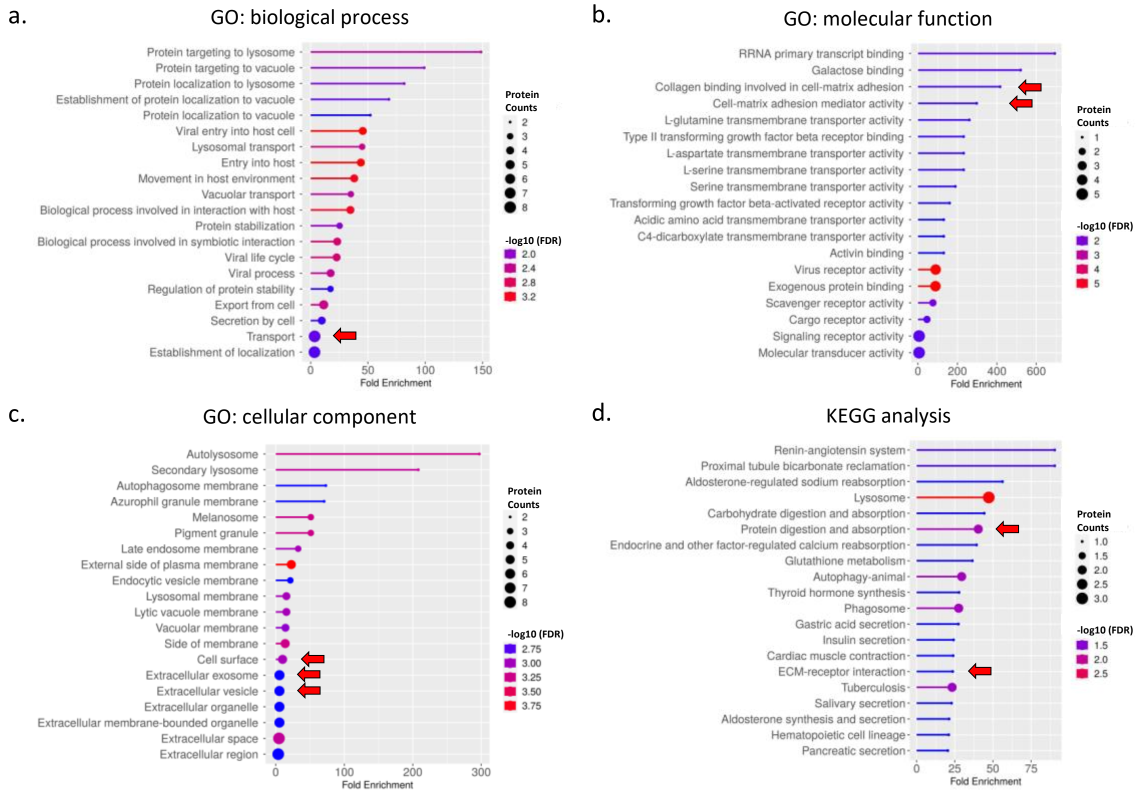

Figure S4. Proteomic cargo annotation and pathway analysis of upregulated and downregulated proteins of Mildly-deg vs. Non-deg, Mildly-deg vs. Deg, and Deg vs. Non-deg groups. A-D. Gene Ontology (GO) within the (**a**) biological process, (**b**) molecular function, and (**c**) cellular component terms and (**d**) the Kyoto Encyclopedia of Genes and Genomes (KEGG) pathway analyses presenting the top 20 annotations. The analyses show (**A**) the upregulated proteins in the comparison between the Mildly-deg and Non-deg samples, (**B**) the upregulated proteins in the comparison between the Mildly-deg and Deg samples, (**C**) the downregulated proteins in the comparison between the Mildly-deg and Deg samples, and (**D**) the upregulated proteins in the comparison between the Deg and Non-deg samples. GO and KEGG false discovery rate (FDR) cut-off: 0.1. Annotation/pathway size: minimum 2, maximum 5000. Circle size refers to the protein counts, colours represent the logarithmic scale of the FDR ($-Log_{10}$ (FDR)), and the x-axis describes the fold change of DEPs involved in the enriched GO annotations or KEGG pathways. The red triangles highlight the annotations/pathways of interests.

The functional annotations of Gene Ontology (GO) terms related to the upregulated proteins comparing Mildly-deg and Non-deg samples were associated with a positive regulation of lipoprotein lipase activity in the biological process term (**Figure S4A (a)**), glycosaminoglycan binding in the molecular function term (**Figure S4A (b)**), and ECM and EV in the cellular component term (**Figure S4A (c)**). Furthermore, the Kyoto Encyclopedia of Genes and Genomes (KEGG) pathway analysis showed an association with multiple metabolic pathways and metabolic homeostasis, such as the PPAR signalling pathway (**Figure S4A (d)**). Comparing DEPs in the Mildly-deg to Deg samples, the GO functional annotation of the upregulated proteins showed an association with positive regulation of lipoprotein lipase activity as well in the biological process term (**Figure S4B (a)**), ECM structural constituent and collagen and glycosaminoglycan binding in the molecular function term (**Figure S4B (b)**), as well as ECM and EV in the cellular component term (**Figure S4B (c)**). The KEGG pathway analysis showed an association only with cholesterol metabolism (**Figure S4B (b)**). The GO functional annotation analysis of the downregulated proteins comparing the Mildly-deg and Deg samples showed an association with transport in the biological process term (**Figure S4C (a)**), cell adhesion molecule/integrin binding in the molecular function term (**Figure S4C (b)**), and EV in the cellular component term (**Figure S4C (c)**). Additionally, the KEGG pathway analysis showed an association with ECM-receptor interaction, protein digestion and absorption, and necroptosis (**Figure S4C (d)**).

Comparing DEPs in the Deg to Non-deg samples, the GO functional annotation of the upregulated proteins showed an association with transport as well in the biological process term (**Figure S4D (a)**), cell-matrix adhesion in the molecular function term (**Figure S4D (b)**), and cell surface and EV in the cellular component term (**Figure S4D (c)**). The KEGG pathway analysis showed an association with also protein digestion and absorption and ECM-receptor interaction (**Figure S4D (d)**).
